# Enhancing the Spinnability of Cellulose-Based Textile Waste by Doping with High Molecular Weight Bacterial Cellulose

**DOI:** 10.1021/acs.biomac.5c02370

**Published:** 2026-03-06

**Authors:** Kaniz Moriam, Crystal E Owens, Laurel Kroo, William Ghann, Jamal Uddin, Leena Pitkänen, Michael Hummel, Gareth H McKinley

**Affiliations:** † Hatsopoulos Microfluids Laboratory, Department of Mechanical Engineering, 174277 Massachusetts Institute of Technology, Cambridge, Massachusetts 02139, United States; ‡ Department of Bioproducts and Biosystems, 14707 Aalto University, Espoo 02150, Finland; § Computer Science and Artificial Intelligence Laboratory, Massachusetts Institute of Technology, Cambridge, Massachusetts 02139, United States; ∥ Center for Nanotechnology, Department of Natural Sciences, 1473 Coppin State University, Baltimore, Maryland 21216, United States

## Abstract

Man-made cellulose fibers from well-managed forestry provide an eco-friendly alternative to polyester and cotton. The Ioncell process converts cellulose-based raw materials into high-quality textiles and offers strong potential for upcycling cellulose-based textile waste. Recycling discarded textiles is challenging because washing and abrasion degrade synthetic and natural fibers, reducing molecular weight and processability. Here, we demonstrate that adding a very small fraction of ultrahigh molecular weight bacterial cellulose enhances the spinnability of textile waste streams dominated by short-chain cellulose. This high molecular weight dopant systematically increases solution extensibility, stabilizing the extension-dominated fiber-spinning process. Viscoelastic stresses in a stable spinline scale with steady extensional viscosity at high strain rates and depend sensitively on chain extensibility. We quantify the enhanced tensile stress differences using capillarity-driven extensional rheometry combined with transient exponential shear rheometry to develop a *spinnability* metric for cellulose/ionic liquid solutions. These findings advance strategies for efficient recycling of postconsumer cellulose textiles.

## Graphical abstract

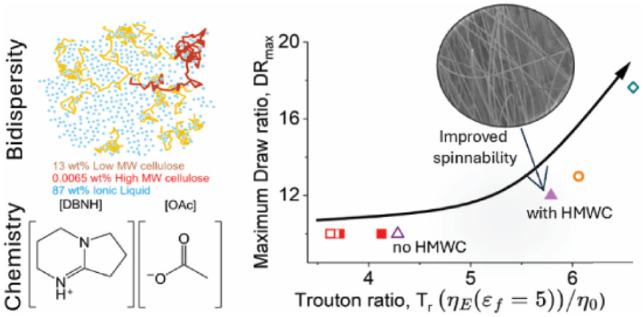

## Introduction

The textile industry is increasingly producing large volumes of clothing to feed *fast fashion* industries,^1^ which tends to encourage the underutilization of materials. Consequently, the global textile market is dominated by cheap synthetic fibers (69%; mainly polyester), followed by cotton fibers (23%).^2^ Synthetic fibers such as polyester are produced from nonrenewable sources at low cost but with a considerable ecological footprint.^3,4^ Furthermore, a recent report showed that current recycling strategies for synthetic textile fibers such as polyester can produce a 10-fold increase in CO_2_ emissions.^5^ On the other hand, although cotton is a renewable resource, its ecological footprint can exceed the adverse effect of polyester due to the unsustainable consumption of water and fertilizers.^3,6^ Collectively, the continuation of such textile production patterns may dominate 26% of the global carbon budget by 2050, which has the potential to eventually increase global warming by 2°C.^7^ Additionally, synthetic textiles are the major source of microfibers or microplastics—emerging global contaminants, polluting land^8^ and the marine environment,^9,10^ which are adversely affecting human and animal health by entering into the food chain.^11–13^

Considering biodegradability and eco-friendliness, man-made cellulose fibers (MMCFs) could at least partially replace synthetic fibers.^14^ MMCFs produced from wood pulps that have been derived from sustainable forestry or other lignocellulosic raw materials, are significantly more sustainable than synthetic fibers and cotton—especially when integrated into pulp production, as demonstrated by extensive life-cycle assessment studies.^15^ Viscose and Lyocell are two major important processes for producing regenerated cellulose fiber.^2,16^ The viscose process has the disadvantage of using large quantities of harmful chemicals, which can pose a threat to the environment and workforce.^17,18^ By contrast, the *N*-methylmorpholine-*N*-oxide (NMMO)-based Lyocell technology is a closed-loop technology, but with the disadvantage that the solvent is unstable under certain process conditions—requiring the addition of a stabilizer.^19–21^ A recently developed variant of the Lyocell process based on ionic liquids (ILs), called the Ioncell process, has overcome the drawbacks mentioned above. Ioncell technology is based on a recyclable superbase-based ionic liquid, which can dissolve cellulose-containing raw materials such as pulp, postconsumer cellulose-based textile materials, or other lignocellulosic substrates in high concentrations to prepare spinnable dopes and subsequently, MMCFs can be produced at scale by dry-jet wet spinning.^16,22,23^

Textile industries can move toward sustainability by recycling existing textile waste rather than creating new ones. Currently, only approximately 1% of textile waste is recycled.^7^ The reuse of postconsumer textiles can effectively reduce environmental pollution caused by dumping textile waste in landfills and incineration. Thus, recycling existing cellulose-based textile fibers is crucial for establishing sustainable textile manufacturing and for promoting a circular economy. Eco-friendly textile production and textile waste recycling can be performed by the newly developed Ioncell technology and similar IL-based spinning processes (such as Kuura fiber,^24^ HighPerCell^25^). As a proof-of-concept, a number of products, including postconsumer cotton^26^ and polyester-cotton blend textiles^6^ were recycled in a batch process. The efficiency of textile waste recycling is limited by the fiber *spinnability*—the ability to spin filaments or fibers from a given complex fluid—as well as the subsequent fiber yield and quality. The spinnability of cellulose solutions is dependent on a multitude of factors, such as the molar mass distribution (MMD) of the raw materials,^27^ the presence of organic and inorganic impurities^28,29^ and the nature of the cellulose-dissolving solvent.^23,29^

Cellulosic materials recovered from consumer waste progressively lose their quality due to washing and abrasion cycles which cause a continuous reduction of the polymer chain length, making it a challenge to successfully recycle them.^30^ Spinning technologies require certain molecular mass distributions of the macromolecules dissolved in the fiber “dope” (i.e., the spinning solution) for efficient and continuous fiber production. Current state-of-the-art technologies rely on blending different materials to adjust the average molar mass and the molar mass distribution. Viscose-based textile wastes are particularly difficult to manage because even newly produced viscose fibers have a low degree of polymerization (DP), which then decreases further during market use. In a recent publication, bacterial cellulose (DP ∼ 1600) was blended with low molecular weight viscose fibers, and the spinnability of the viscose textile waste was empirically found to be improved.^31^

Previous fundamental research has shown that even a very small weight fraction of ultrahigh molecular weight polymer can promote fiber formation of weakly entangled and “nonspinnable” polymer solutions.^32–35^ Recent systematic studies of electrospinning using solutions of synthetic fibers with controlled polydispersity have shown that the spinnability can be systematically tuned by adjusting a new measure of the molecular weight distribution (the extensibility-averaged molecular weight *M_L_*) through the addition of small amounts of a high molecular weight (HMW) additive.^32,36^ In the present work we follow the same approach and use a specific grade of very high molecular weight cellulose known as bacterial cellulose that is derived from bacterial fermentation. We hypothesize that doping textile waste (which is characterized by broad molecular weight distributions of short-chain cellulose molecules that convey poor processability) through the introduction of a very small concentration (less than the critical overlap concentration c*) of a high molecular weight bacterial cellulose (or HMWBC; DP ∼ 4900) will improve the spinnability of the resulting solution.

To systematically explore and understand these changes we use a range of rheological characterization techniques that quantify the linear viscoelastic response, the steady shear viscosity, and the transient extensional viscosity of the cellulose/ionic solutions. To enhance the efficiency of textile waste recycling, it is essential to understand the structure and rheology of cellulose blends in the native solution state that is employed during processing operations, and how it changes with concentration and molecular weight. Here we construct rheological “master curves” that compactly summarize the changes and scaling of cellulosic solution rheology with concentration, temperature and the presence of dilute high molecular weight additives. Specifically, it is known that the extensibility of a bidisperse system varies dramatically with addition of a very small fraction of very long and extensible chains, and this can help stabilize extension-dominated processes such as filament thinning or fiber spinning.^37,38^ However, such changes can be very difficult to measure in shear deformations. The presence of vorticity and molecular tumbling means that the dilute amount of the high molecular weight species present in the spinning dope does not contribute substantially to the total stress that develops in the sheared system.

However, the characteristic viscoelastic stresses generated in a spinline can be accurately assessed by measuring the steady-state extensional viscosity of flexible and semiflexible polymer solutions at high strain rates.^32,37^ It is thus critically important to investigate the elongational or extensional flow of cellulose-IL spinning solutions or “dopes” because industrial dry-jet fiber spinning processes involve a predominantly extensional mode of deformation before the thinning and elongating fluid filaments are formed into regenerated fibers by the process of solvent exchange. Understanding the transient extensional properties of macromolecular solutions can provide insights into the compositional and process parameters that prevent the breakup of filaments during spinning and promote spinnability of the solution.^39,40^ Capillary Breakup Extensional Rheometry (CaBER) analysis has been performed in the past for a wide variety of cellulose-IL solutions,^39,41,42^ however, very few studies have directly connected measurements of the transient extensional rheology with the ensuing fiber spinning processes.

In fiber-spinning operations the characteristic strain rate in the thinning filament varies spatially along the spinline and can be controlled by varying the draw ratio.^41,43^ By contrast, in the CaBER technique a self-similar balance is established between the squeezing action of capillarity and the viscoelastic tensile stresses that develop in the thinning filament.^38,44^ As a consequence, it is not possible in the CaBER approach to systematically vary the imposed strain rate imposed to the sample. The filament stretching extensional rheometer (FISER) has traditionally been used as an alternate method for determining the transient extensional viscosity across a range of strain rates.^45^ However, a notable issue is the nonuniformity of the strain rate produced along the device, leading to spatially inhomogeneous deformation and the introduction of mixed shear and extensional kinematics which necessitate advanced feedback control strategies in order to measure a true extensional viscosity.^46,47^ An alternate approach is to employ a spinline rheometer (SLR), however in such devices, the residence time of polymers in the spinline can vary substantially depending on the specific streamline they follow. This streamline-dependent strain experienced by material elements can obfuscate the resulting stress growth dynamics and lead to a multivalued “spinline viscosity function”.^48^

In an effort to address some of these practical challenges, Doshi and Dealy (1987) proposed an alternative approach to probing extensional stress growth in polymeric samples undergoing strong transient extensional processes using a bespoke deformation protocol called *Exponential Shear (ES) flow*.^49^ In an exponential shear flow, the shear rate imposed on the sample is programmed to increase exponentially over time to replicate the kinematics of a material element in planar extensional flow.^50^ This strong time-dependent shear flow results in exponential growth of the principal stretch (or elongation ratio) of material elements in the sample, thus mimicking the kinematics of true extensional flow. Exponential Shear (ES) offers several advantages over other extensional rheometric methods. First, it allows the generation of extension-dominated kinematics using commonly available shearing devices. Moreover, direct computation of the evolving principal stress difference in the sample becomes feasible by combining the torque and normal force measurements, and the imposed deformation rate remains uniform throughout the sample. Earlier efforts at measuring tensile stress growth in entangled polymer solutions undergoing exponential shear^51^ were frustrated by the limited total extensional strains that could be achieved, but recent hardware and software advances in modern rheometric instrumentation have relieved this constraint.^52^ The exponential shear rheometry approach thus provides a promising alternative to traditional filament stretching devices for studying the transient extensional rheological behavior of polydisperse macromolecular solutions such as spinning dopes, as well as connecting such measurements to observations of spinnability as the composition is systematically varied. To our knowledge, this method has not yet been used to date in the study of cellulose-ionic liquid systems.

In the present study, we investigate the spinnability of different cellulose solutions and the sensitivity to “doping” with high molecular weight bacterial cellulose as a dilute processing additive, using an array of established techniques (dry-jet wet spinning production of fibers, small-amplitude oscillatory shear measurements over a range of temperatures and concentrations, CaBER measurements of the transient extensional rheology) as well as through exponential shear (ES) rheometry. We employ this latter technique with a specific focus on the influence of concentration, the presence of high molecular weight bacterial cellulose as an additive, and variations in the degree of polymerization (DP) on the transient extensional stress growth. Since the extensional stresses depend on the molecular weight distribution of the stretched chains and their extensibility, we can also hypothesize that doping polydisperse solutions with high molecular weight bacterial cellulose will augment the transient extensional viscosity and the viscoelastic relaxation time of postconsumer cellulose/IL solutions. Along with the above-mentioned textile waste samples (with and without addition of a bacterial cellulose dopant), we also explore a range of other spinning solutions: a low-concentration (5 wt %) cellulose pulp dissolved in IL, a medium concentration solution (8 wt %), and a standard spinning dope (13 wt % cellulose pulp in IL). Additional compositional variations can be introduced by incorporating different percentages of bacterial cellulose into the low-concentration cellulose solution. Our goal is to systematically explore how to enhance the spinnability of low-quality postconsumer textile waste.

## Materials and Methods

### Materials

Preconsumer viscose fibers (Kelheim Fibers GmbH), prehydrolysis kraft pulp (Enocell; Stora Enso), bacterial cellulose (Center of Biological Engineering, University of Minho) were used as received. Equimolar amounts of 1,5-diazabicyclo[4.3.0]­non-5-ene (99% Fluorochem, UK) and acetic acid (100% Merck, Germany) were used to synthesize the ionic liquid 1,5-diazabicyclo[4.3.0]­non-5-ene acetate [DBNH]­[OAc] for the dissolution of cellulose.

### Dope Preparation

The spinning dopes were prepared using a vertical kneader system. Air-dried cellulose was added to the molten ionic liquid (IL) while premixing manually with a Teflon spatula. The mixture was then kneaded for 1.5 h at 10 rpm and 80 °C under 30–50 mbar vacuum to avoid inclusion of air bubbles. The solution was subsequently press-filtered using a hydraulic press at 2 MPa and 90 °C through layered filter mesh (GKD Ymax2, 5 μm nominal, Gebr. Kufferath AG, Germany) to remove residual undissolved particles.

The definitions of all samples, with and without HMWBC addition, are listed in Table S2. Due to the high molecular weight of HMWBC, increasing its concentration toward or above the estimated overlap concentration (c*) resulted in sharply increased dope viscosity and dissolution heterogeneity during preliminary trials. Therefore, all formulations investigated in this study were prepared in the dilute-to-suboverlap regime (*c* ≪ c*) to ensure homogeneous dopes and reproducible spinning behavior.

### Intrinsic Viscosity Measurement

The intrinsic viscosities of the cellulose were measured according to the standard described in SCAN-CM 15:88.^30,31,53^ Dry samples were each dissolved using cupriethylenediamine aqueous solution (CED, CAS:14552-35-3). Consequently, about 250 mg of each sample was weighed into plastic bottles with five copper rods and further filled with 25 mL deionized water. The plastic bottles were shaken for 30 min in a Kauko Lehtinen shaking device, at room temperature. Then, 25 mL of CED was added (yielding a final concentration of around 0.005 g/mL) and again shaken for 30 min, to facilitate cellulose dissolution. All samples were then placed in a 25 °C water bath (Mistral Multistirrer) before measurement for temperature equilibration. The intrinsic viscosity of all solutions was measured using a regular capillary viscometer (Schott Geräte, AVH 350, Hofheim, Germany) with a capillary diameter of 0.63 mm. Then, the determined intrinsic viscosity [η] was used to estimate the degree of polymerization (as described in SCAN-CM 15:88):^30,53^

1 $$DP={\left(\frac{\left[\eta \right]}{Q}\right)}^{1/a}$$

where the *Q* and *a* parameters are defined as follows:^54^ when DP< 950, *Q* = 0.42 mL/g and *a* = 1; when DP *>* 950, *Q* = 2.28 mL/g and *a* = 0.76; the SI units of [η] are mL/g.

### Size-Exclusion Chromatography (SEC) for Molar Mass Distribution Measurement

The molecular weight distribution was measured with gel permeation chromatography (GPC) using a Dionex Ultimate 3000 HPLC system equipped with a Shodex DRI (RI-101), and a Viscotek/Malvern SEC/MALS 20 multiangle light scattering (MALS) detector.^55^ About 0.05 g of each pulp/sample was subjected to a solvent exchange process, consecutively using water, acetone, and DMAc (4 mL of each reagent). The samples were immersed in the solvent overnight for effective activation. After solvent exchange, cellulose samples were dissolved in LiCl/DMAc and stirred overnight, to ensure full dissolution. The samples were then diluted (from 90 g/L of LiCl to 9.0 g/L of LiCl). The diluted samples were filtered using 0.2 μm filters into plastic vials before measurements. 100 μL of each sample solution was placed into the four-column system (PLgel MIXED-A), operating at a flow rate of 0.75 mL/min. A narrow polystyrene standard (*M*_*w*_ = 96,000 g/mol, *Đ* = 1.04, refractive index increment, ∂*n*/∂*c* = 0.146 mL/g) was used to obtain the detector constants for MALS and DRI, whereas a polydisperse polystyrene sample (*M*_*w*_ = 248,000 g·mol^–1^; *Đ* = 1.73) was applied to test the calibration of the detectors. A refractive index increment of ∂n/∂c = 0.136 mL·g^–1^ was used for celluloses in 0.9% LiCl in DMAc.^56^ After GPC analysis, the weight-average molar mass (*M*_*w*_), number-average molar mass (*M*_*n*_), dispersity (*Đ = M*_*w*_/*M*_*n*_), and the proportion of long cellulose chains (DP > 2000), and short cellulose chains (DP < 100) were obtained.

### Extensibility-Averaged Molecular Weight

The extensibility-averaged molecular weight *M_L_* has been proposed as a measure that more accurately captures the effect of molecular polydispersity on the extensional stress developed in a polymer solution undergoing a uniaxial extensional flow by weighting the individual contributions of polymer molecules of different chain length to the total tensile stress difference generated in an extensional flow.^32^ Unlike conventional measures of the average molecular weight of a polydisperse system, *M_L_* is specifically tailored to reflect the impact of chain length on the extensibility or deformability of fibers under strong extensional (stretching) kinematics.

In mathematical terms, *M_L_* is calculated using a weighted sum or distribution function that weights more strongly the chains that contribute more to the total extensional stress in the filament. This means that longer chains, which are more extensible and contribute more to the total tensile stress difference, have a greater influence on *M_L_* than shorter chains. The extensibility-averaged molecular weight is thus a more accurate representation of how trace amounts of high molecular weight additives impact fiber strength and elongation during fiber formation in an extension-dominated process such as fiber spinning, dry-jet wet spinning or electrospinning.^32,36^

We compute the extensibility-averaged molecular weight, *M_L_* using the following equation which has been introduced previously.^32,36^

2 $$M_{L}={\left(\sum w_{i}{M_{i}}^{1+\nu }\right)}^{1/1+\nu }$$

Here, *w_i_* is the weight fraction of species *i*, *M_i_* is the molecular weight of that polymer species and ν is the solvent quality parameter of the chains in solution.

The value of this average is clearly influenced by the solvent quality ν, as the interactions between polymer and solvent impact the initial polymer chain conformation and, thus, its maximum extensibility. From Zimm theory for dilute solutions of flexible chains incorporating hydrodynamic interactions, the intrinsic viscosity is found to depend on the unperturbed coil size as [η] ∼ (*M_v_*)^3ν–1^ where *M_v_* is the *viscosity-averaged molecular weight.* For flexible polymers in good solvents or near-theta conditions, typically ν ≈ 0.5–0.6.^57^ For semirigid polymers like cellulose with a higher persistence length, ν ≈ 0.6–0.75 in good solvents, reflecting their greater swelling due to increased rigidity. In semirigid polymers such as cellulose, the swelling behavior follows a modified scaling law, but the approximation *a* ≈ 3*ν* – 1 between the Mark–Houwink parameter and the solvent quality remains useful for predicting cellulose chain dimensions in solution.^58^ This interplay of molecular weight, solvent quality, and chain rigidity is essential for controlling polymer extensibility and spinnability in cellulose fiber production processes.

To calculate the values of *M_L_* for each spinning dope we have used the viscosity-averaged molecular weight *M_v_*, and the Mark–Houwink parameter *a* values obtained from the intrinsic viscosity measurement (Table S1). Intrinsic viscosity and Mark–Houwink parameters were determined in cupriethylenediamine (CED) aqueous solution, a standard reference solvent for cellulose; these parameters were used to obtain comparative extensibility-averaged molecular weights for rheological analysis rather than absolute molecular weights in the ionic liquid.

An additional complication is that in dry-jet wet spinning a range of different total polymer concentrations (denoted *c*) are also commonly used. To be able to intercompare the extensibility of different composition solutions we recognize that the weight fraction of a particular species *i* in eq 2 is *w_i_* = *c_i_*/*c*. Substituting this expression in eq 2 and rearranging, the concentration-dependent extensibility average molecular weight, is obtained as follows

3 $$cM_{L}^{1+\nu }=\left(\sum c_{i}M_{i}^{1+\nu_{i}}\right)=c\left(\sum w_{i}M_{i}^{1+\nu_{i}}\right)$$

The values of *M_L_* and *c* $M_{L}^{1+\nu }$ for the cellulose solutions used in this work are reported in Table 1.

**Table 1:** Summary of the Extensibility Average Molecular Weight Parameters $M_{L}^{1+\nu }$ , $cM_{L}^{1+\nu }$ for the Different Samples Used in This Study

Sample name*^a^	***a***	ν	*c* (w/w)	*M_L_* (kDa)	${M_{L}}^{1+\nu }$ **^b^	*c* ${M_{L}}^{1+\nu }$ **^b^
E3	0.76	0.58	0.03	161	3068	92
E5			0.05	161	3068	153
E5B0.0005			0.0505	172	3419	173
E5B0.0025			0.0525	214	4826	253
E8			0.08	161	3068	245
E13			0.13	161	3068	399
V13	1	0.67	0.13	70	1206	157
V13B0.0065			0.1365	81	1549	211

a The naming convention of the samples is described in detail in Table S2.

b ** For calculating $M_{L}^{1+\nu }$ and $cM_{L}^{1+\nu }$ of V13B0.0065, the solvent quality parameter ν (Table S1) for the dominant cellulose content (viscose waste) was used.

### Dry-Jet Wet Spinning

The cellulose solutions (dopes) were spun using a monofilament spinning unit (Fourné Polymertechnik).^31,59,60^ The diameter of the spinnerete capillary was 100 μm; with an air-gap of 1 cm, and extrusion velocity 2.1 m·min^–1^. The draw ratio *D_R_* was controlled by varying the velocity of the take-up wheel (*godet*).

### Fiber Testing

The tensile properties (tenacity (cN/tex); elongation at break (%) and linear density (dtex)) of the spun fiber samples were measured in the conditioned state using a Favigraph device (Textechno, Germany). Prior to measurement, the fibers were conditioned at 20 ± 2 °C and relative humidity of 65 ± 2% overnight. For each measurement, 20 individual fibers were measured using a 20 cN load cell and 20 mm gauge length.

### Shear Rheology

The shear rheology was analyzed on a Discovery Hybrid Rheometer 3 (TA Instruments, New Castle DE) using an aluminum parallel-plate geometry (40 mm plate diameter; geometry gap 0.5 mm). Measurements of the exponential shear rheology were performed using a controlled strain ARES-G2 (TA Instruments, New Castle DE). A Peltier heating plate was used on both rheometers to accurately control the sample test temperatures.

### Extensional Rheology

Measurements of the transient extensional rheology were performed using a customized Capillary Breakup Rheometer (CaBER).^61^ The cellulose solutions were placed between two coaxial plates (diameter 6 mm) with an initial gap separation *H*_0_ = 10 mm. During the measurement, the plates are separated in a step-strain manner (with an imposed Hencky strain corresponding to log­(*H_f_*/*H*_0_) = 1.35). The temporal evolution of the filament diameter (Figure 1) was captured by high-speed camera (Phantom M320s; Vision Research Inc.) at 50 to 100 frames per second and a spatial resolution of 14 μm/pixel. After an axial step displacement separates the two plates of the CaBER device to a strain ε_0_, an unstable liquid bridge of the viscoelastic test liquid is formed between the plates, which then becomes progressively thinner with time under the action of capillary pressure.^41^ A high-speed video is recorded, and the evolution of the midpoint radius of the thinning viscoelastic fluid filament with time is measured by an edge-detection algorithm. The extensional relaxation time of the polymer solution is calculated from an elastocapillary balance on the thinning liquid filament, which becomes appropriate on small length scales.^44,62,63^ The evolution of the minimum filament radius with time *R­(t)* in the elastocapillary region is given by

4 $$\frac{R\left(t\right)}{R_{0}}\approx {\left(\frac{GR_{0}}{2\Gamma }\right)}^{1/3}\exp\left[-t/3\lambda_{E}\right]$$

**Figure 1:**
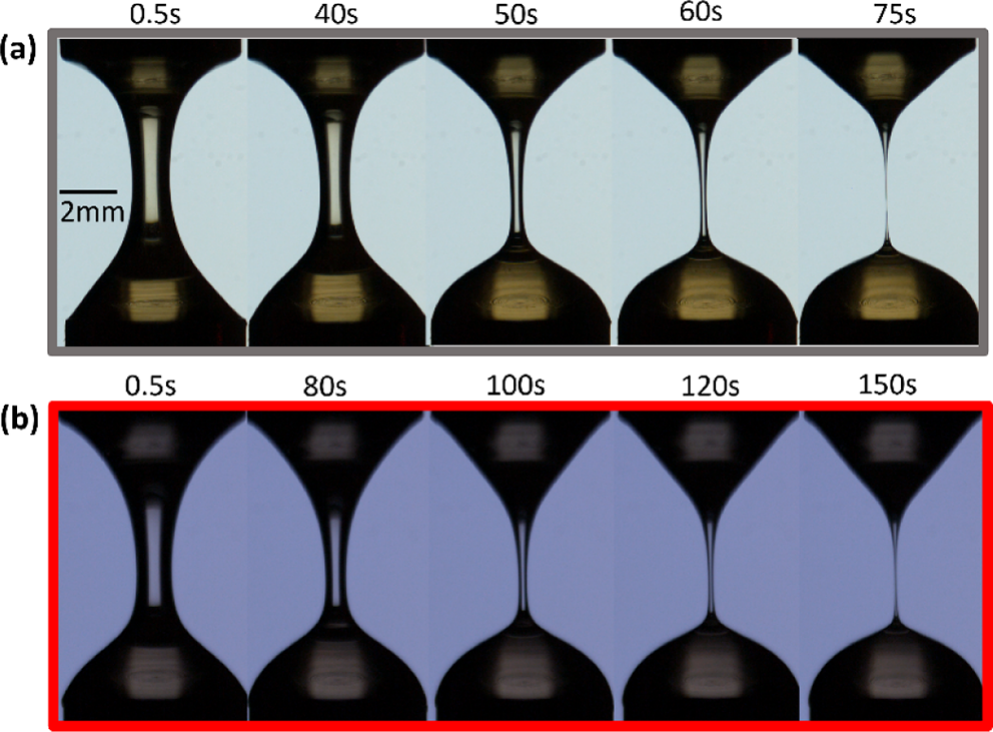
Snapshots showing the capillarity-driven thinning profiles for the selected cellulose solutions: gray outlined box (a) shows solution E5 (without bacterial cellulose), with concentration extensibility parameter $cM_{L}^{1+\nu }$ = 153; (b) red outlined box shows solution E5B0.0025 (with bacterial cellulose) with concentration extensibility parameter $cM_{L}^{1+\nu }$ = 253.

The initial plate radius is *R*_0_ = 3 mm, the surface tension Γ, is taken here as 0.035 N/m,^64^ *G* is the viscoelastic modulus of the polymer solution, and λ*_E_* is the extensional relaxation time.

Elastic and capillary stresses dominate the overall stress balance within the thinning filament in the elastocapillary regime. Polymer stretching, orientation, and conformational changes contribute an additional tensile elastic stress difference Δσ = η*_E_* ε̇, in the neck that opposes the capillary stress, Γ/*R*(*t*). The strength of the extensional flow that drives the progressive thinning of the neck can be quantified in terms of a time-varying extension rate, ε̇(*t*),

5 $$\varepsilon ̇=-2\frac{d\;\ln R\left(t\right)}{dt}$$

The apparent extensional viscosity can then be evaluated using the following formula,^43^

6 $$\eta_{E,\mathrm{app}}^{+}=\frac{\Gamma }{\varepsilon ̇R\left(t\right)}=\frac{\Gamma }{-2dR\left(t\right)/dt}$$

Though the extensional viscosity, η*_E_*, is only a factor of 3 times larger than the zero shear viscosity for Newtonian fluids, the apparent Trouton ratio for non-Newtonian polymeric solutions, defined as Tr = $\eta_{E,\mathrm{app}}^{+}/\eta_{0}$ can become orders of magnitude greater than Tr = 3.^65^

### Time–Temperature and Time–Concentration Superposition

The small amplitude oscillatory shear (SAOS) results were reduced through time superposition techniques using the rheometer software (TRIOS v5.1.1.46572) to collapse the measured values of the complex viscosities and moduli at different experimental conditions onto a master curve.^41^ To reduce multiple variables, time–temperature superposition (tTS) and time–concentration superposition (tCS) were both employed. Time–temperature superposition master curves were constructed using a reference temperature close to the spinning temperature. In each tTS superposition curve, the data at 50 °C was chosen to be the reference, and for tCS, the lowest concentration was used as the reference condition. The shift parameters were calculated using TRIOS software using the following equation:

7 $$a_{[x]}=\frac{G\left(x\right)}{G\left(x_{0}\right)}\cdot \frac{x_{0}}{x}\cdot \frac{\rho_{0}}{\rho }$$

Where, *G* generically represents either *G*′ or *G*″, the measured storage or loss modulus and [*x*] generically denotes the variable being superposed. Here ρ_0_ and ρ­(*T*) are the material densities at reference temperature *T*_0_ and temperature *T*, where for the small range of absolute temperatures used in the present study we take ρ*(T = T*_0_ *)/*ρ­(*T*) = ρ_0_/ρ­(*T*) ≈ 1. When superimposing results at different temperatures, the activation energy for flow, *ΔH*, can be obtained from horizontal shifting according to the Arrhenius equation,

8 $$a_{T}=\exp\left[\frac{\Delta H}{R}\left(\frac{1}{T}-\frac{1}{T_{0}}\right)\right]$$

where *R* = 8.314 J/mol·K is the universal gas constant.

### Exponential Shear Rheology

A standard, strain-controlled torsional rheometer was used to perform the exponential shear experiments (TA Instruments ARES G2). The sample is loaded between the cone and plate and a bespoke shear strain profile is then applied to generate a strong extensional flow. This instrument can impose an arbitrary, programmable input strain as a function of time, controlled by the direct drive motor attached to the lower fixture that is in contact with the sample. At the upper fixture, high-resolution rebalance sensors report both the torque and the axial normal force, which are generated by the test sample in response to the imposed strain. These torsional and axial forces may be converted into stresses, a process which is performed by the instrument software (TRIOS v5.1.1.46572) using conversion factors that are dependent on the specific rheometric test attachment used. Importantly, the shear stress (*σ_yx_* (*t*)) and the normal stress difference (*N*_1_(*t*)) are measured simultaneously but independently. In these experiments, we chose to use a 40 mm cone-and-plate test geometry to maintain a spatially homogeneous stress distribution throughout the sample.

Consistent with prior experimental papers using this method,^49^ the input strain is given by,

9 $$\gamma \left(t\right)=2\;\sinh\left(\alpha t\right)=e^{\alpha t}-e^{-\alpha t}$$

where α is an effective Hencky strain rate (ε̇), with units of inverse seconds. It is noted that for the duration of these experiments, this rate is a constant set by the user (similar to filament-stretching rheometers; but quite distinct from CaBER or dripping-on-substrate (DoS) methods). For most of the results presented we chose a value of α = 7 s^–1^, up to a final Hencky strain of ε*_f_* = 5 (corresponding to a final time given by *t_f_* = ε*_f_*/α). This choice of α = 7 s^–1^ was selected to mimic the strong stretching rates relevant to the processing conditions of fiber spinning. It is notable that the majority of prior studies using exponential shear probe the mechanics of this transient stretching flow at far more moderate Hencky strain rates (α = 0.1–2 s^–1^) and Hencky strains (3 < ε*_f_* <4).^50^ Only recently did the field begin to use exponential shear to probe materials at strain rates relevant to the fiber-spinning industry. Specifically, the reader is referred to the study by Kroo et al. (2025)^52^ for a detailed analysis of kinematic limitations of exponential shear, and to our knowledge, the first example of exponential shear performed on viscoelastic fluids in the regime of large strain amplitude and high strain rates relevant to the present study.

To compute the transient extensional properties of the fluid from the exponential shear experiments, we first compute the principal stress difference in the direction of the principal stretch. We combine the normal stress difference and the shear stress to compute the total principal stress difference, (Δσ) along the axis of material stretch, which is given by $\Delta \sigma \left(t\right)=\sqrt{4\sigma_{yx}^{2}\left(t\right)+N_{1}^{2}\left(t\right)}$ . As discussed in detail by Kroo et al. (2025),^52^ the time-varying vorticity that is generated in this transient shear flow does not strongly influence the dynamics of the principal stress growth when performed on highly viscoelastic fluids and at high stretching rates. Exponential shear measurements were performed directly at the spinning temperature to ensure relevance to processing conditions.

### Field Emission Scanning Electron Microscopy (FESEM) Imaging

The morphology of the spun fibers was analyzed using field emission scanning electron microscopy (FESEM; JSM-7100FA JEOL USA, Inc.) To ensure electrical conductivity, the samples were sputter-coated with gold using a JEOL Smart Coater under the following conditions: a sputtering pressure of 4 Pa, a working distance of 20 mm, and a sputtering time of 1 min, which produced a coating thickness of approximately 5 nm. The secondary electron images were taken at a 1.5 kV operating voltage at ∼3k magnification.

## Results and Discussions

### Shear Rheology

We have used the 5 wt % prehydrolysis kraft (PHK) dissolving pulp-ionic liquid (IL) solution as the standard low-viscosity solution (defined as sample E5 in Tables S1 and S2). Figure 2a shows the complex viscosity of the 5 wt % PHK solution in ionic liquid as a function of angular frequency at temperatures varied from 40 to 80 °C. Increasing the temperature lowers the complex viscosity, and the viscosity curve shows frequency-thinning behavior at higher oscillatory frequencies. Various authors have reported similar behavior for cellulose-IL solutions previously.^39,41^ The method of reduced variables^66^ was used to generate the master curves from the measured complex viscosity data (Figure 2, Figures S1–S6). All the solutions, including PHK-pulp, PHK pulp doped with bacterial cellulose (BC) and BC-incorporated preconsumer viscose-based solutions, behave in a similar manner, and master curves were constructed for each sample.

**Figure 2:**
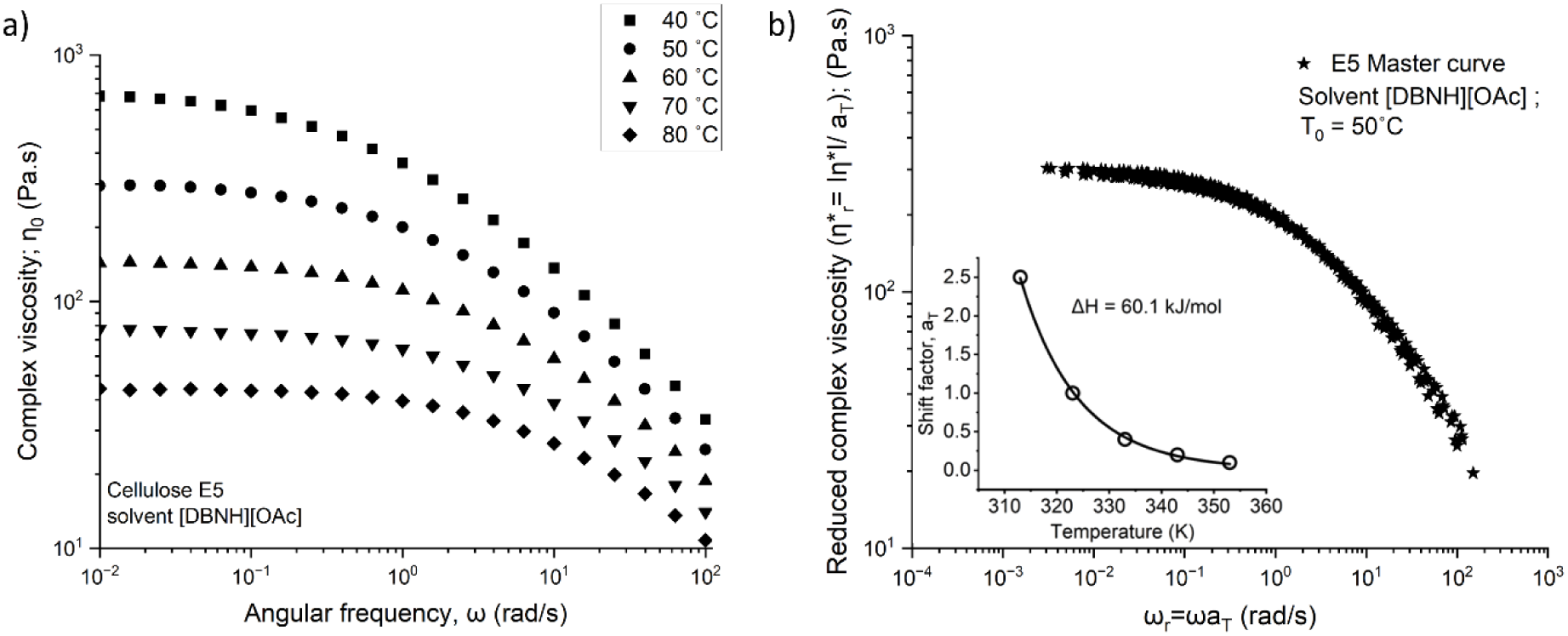
(a) Complex viscosity as a function of angular frequency at different temperatures (40 ≤ *T* ≤ 80 °C) for 5 wt% PHK pulp (E5), (b) the SAOS master curve obtained via time–temperature superposition (tTS) referenced at *T*_0_ = 50 °C. The shift factors *a_T_* (*T_j_*, *T*_0_) are plotted as an inset.

The shift factors showed considerable dependence on temperature and are well described by the Arrhenius eq (Figure 2 and Figures S1–S6). From the slope of the Arrhenius plot, and eq 8 the activation energy for flow was calculated for each solution concentration. The Arrhenius model reflects exponential dependence of the viscosity and other dynamic properties of simple and complex liquids at elevated temperatures far above the glass transition temperatures.^67^ This model applies well at higher temperatures, where activation energy barriers for local chain rearrangement dominate the flow behavior of the material.

The activation energy is influenced by the molecular structure of the polymer chains, the intensity of the interchain interactions between molecules and the solvent, as well as the bulkiness and rigidity of the side chains in the molten state.^68^ The activation energy of polymer solutions generally increases with increasing concentration. Previous works have reported a similar trend for cellulose-IL solutions^67^ and cellulose-NMMO solutions.^68^ The bacterial cellulose-incorporated sample shows a higher activation energy than sample E5, and the value of Δ*H* increases again for the concentrated system (E13) reflecting the increased segmental concentration in solution (see Figures S1, S2 and S6, and Table S3). The resulting tTS master curves for all three polymer concentrations are shown in Figure 3a

**Figure 3:**
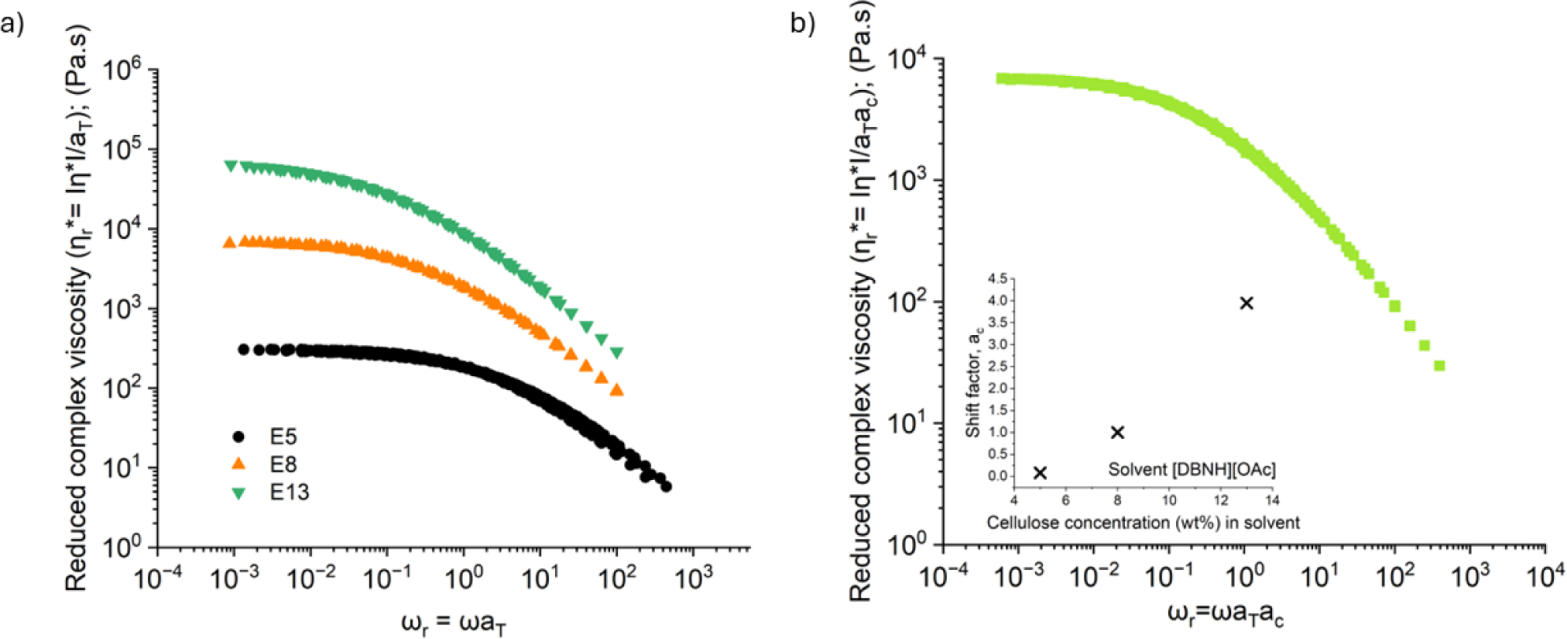
(a) The SAOS master curves obtained via time–temperature superposition (tTS) for the cellulose solutions at different concentrations, 5 wt %, 8 wt %, and 13 wt %, referenced to 50 °C. (b) The SAOS master curve obtained via time–concentration superposition (tCS) for all cellulose solutions dissolved in [DBNH]­[OAc] referenced at 8 wt % and *T*_0_ = 50°C.

Next we investigate how total cellulose concentration also increases the viscoelasticity of the cellulose/ionic liquid solutions using time–concentration superposition (tCS).

The coil overlap concentration c* is the concentration at which polymer chains in a solution start to overlap. Below c*, individual polymer chains are isolated and do not interact significantly with each other. In this regime, viscosity increases linearly with concentration.^69^ Above c*, the polymer chains begin to interpenetrate and overlap as the system enters the semidilute regime. The entanglement concentration, *c*_e_ is the concentration at which polymer chains in a solution become entangled with each other. Above *c*_e_, the solution is in the semidilute entangled regime, and the viscosity of the solution increases more rapidly with concentration compared to the unentangled semidilute regime, typically following a power law scaling with a higher exponent.^70–72^ From Figure 3a it is evident that the time–temperature master curves for each concentration of cellulose-[DBNH]­[OAc] are self-similar and can be superposed using a concentration-dependent shift factor *a_c_* (*c*). We pick the *c* = 8 wt % curve as the reference condition and construct the supermaster curve shown in Figure 3b. The inset figure shows that the shift factor increases faster than linearly with concentration. Previous research has shown that the zero shear viscosity follows a power-law dependence on concentration for cellulose-[DBNH]­[OAc] solutions, i.e., η_0_ ∝ *c^n^*, for PHK pulp cellulose solutions above a coil overlap concentration c* > 0.48 wt %.^64^ In semidilute and concentrated regimes, hydrodynamic interactions between polymer chains become significant, contributing to a nonlinear increase in viscosity with concentration. For biological macromolecules, the power law exponent varies depending on the polymer, solvent, and specific conditions, reflecting the complexity and nonlinearity of biopolymer solution behavior in this regime.^73^ In the present study, we find that the power law variation in the viscosity for different concentrations of PHK pulp solution in [DBNH]­[OAc] at 40 °C shows an exponent of *n ≈* 4.4 (Figure S7a). With increasing concentration, the dynamics of the polymer chains become slower due to entanglements, and the solutions exhibit increasingly viscoelastic behavior.

A complementary approach for understanding the small but systematic changes in the shear rheology of these entangled solutions with addition of small amounts of high molecular weight additives is through the intrinsic viscosity [η] which will be influenced by the rigidity, molecular weight, and molecular weight distribution of the high molecular weight species.^73^ To observe the effects of the HMWBC dopant on the intrinsic viscosity coefficient of the cellulose-IL matrix, in Figure S7b we plot the relative viscosity η*_r_* = η­(*c* ^dopant^)/η_0_(*c*) as a function of dopant concentrations (over the range of all tested concentrations). The increase in the relative viscosity can be described according to the following equation based on the polynomial expansion proposed in the literature.^74^

10 $$\eta_{r}=\frac{\eta_{0}\left(c^{\mathrm{dopant}}\right)\;(\mathrm{cellulose matrix with dopant})}{\eta_{0}\left(c\right)\;(\mathrm{cellulose matrix alone})}=\left\{1+\left[\eta \right]c^{\mathrm{dopant}}+...O{\left(c^{\mathrm{dopant}}\right)}^{2}\right\}$$

The relative viscosity data show some scatter but increase almost linearly with increasing concentration of the bacterial cellulose dopant. The calculated value of the viscosity increment is [η] = 546 ± 40 mL/g, which represents the (large) relative increase in viscosity expected per solute molecule of bacterial cellulose added to the [DBNH]­[OAc] at 50 °C.

### Evolution of Linear Viscoelastic Properties with High MW Additives

At low frequencies, the complex viscosities of the additive-containing dopes differed notably from those without additive but became almost indistinguishable at higher frequencies at a specific temperature (Figure 4a,c). The curves are still self-similar and can once again be superposed to produce new supermaster curves. However, the presence of the dopant results in enhanced values of the concentration shift factors (Figure 4b,d).

**Figure 4:**
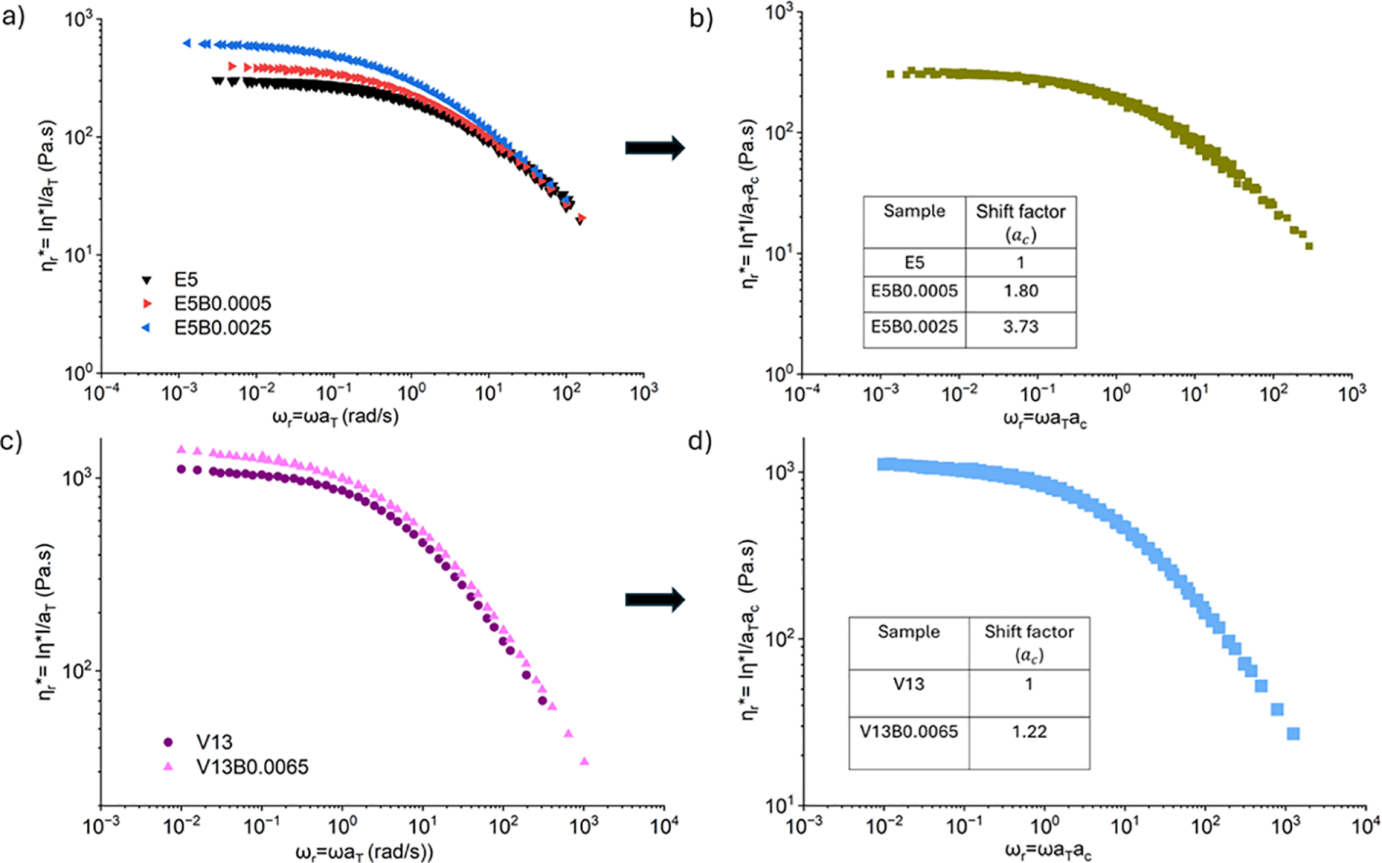
SAOS master curves showing the magnitude of the complex viscosity as a function of frequency for (a) PHK pulp solution in IL with, and without, HMWBC constructed via tTS at the spinning temperature of 50 °C, (b) tCS for the entangled PHK pulp solutions with or without additives, referenced to 50 °C and 5 wt %, (c) time–temperature master curves for preconsumer viscose-based cellulose-IL solution with, and without, HMWBC, (d) tCS referenced at 50 °C and 13 wt %.

The enhanced magnitude of the complex viscosity at low frequencies arises from the presence of the dilute dispersion of high molecular weight bacterial cellulose in the semidilute entangled cellulose matrix. At higher frequencies, the response is predominantly governed by the plateau modulus of the cellulose matrix, leading to very similar values of the dynamic viscosities (Figure S8). Because of the self-similar forms of the viscoelastic master curves, this viscosity enhancement at low frequencies will also be manifested through a corresponding increase in the longest relaxation time in the material, arising from the changes in the molecular mass distribution of the doped system.^75^ For a given set of spinline conditions, this increase in the dominant relaxation time of the doped solutions will be reflected in higher values of the Weissenberg number, and enhanced nonlinear viscoelastic effects during subsequent fiber spinning operations.

### Cellulose Fiber Spinning

The spinnability of a cellulose solution refers to its ability to stably form and stretch a fluid filament in the air gap without breaking over a specified time period. This ability to stretch is measured by the maximum draw ratio (DR) attainable, which is the ratio of the take-up velocity to the extrusion velocity. Previously, the spinnability of dope has been classified based on the maximum stable draw ratio achievable, and subdivided into the following categories for solutions with a concentration of cellulose of 13 wt % or higher:^26,28^ DR < 2 nonspinnable, 2 ≤ DR < 8 poor, 8 ≤ DR ≤ 18 good, and DR > 18 means excellent spinnability. However, lowering the cellulose concentration can also affect spinnability. For a low-concentration dope, the stable fiber diameter is thinner compared to a high-concentration dope, and the achievable draw ratio is also typically lower. We have used a lower concentration of PHK pulp (5 wt % in IL) as a standard solution to mimic the lower molecular weight of the viscose solution. Regardless of the presence of bacterial cellulose, our initial experiments showed that the maximum draw ratio attainable with the 5 wt % solutions was DR ≈ 10, and the linear densities of the resulting fibers were more or less identical (0.8–0.9 dex, Table 2). This is despite the clear differences in the linear viscoelastic properties of the solution (Figure 4a,c). For this relatively high molecular weight cellulose material (cf. Table: *M_L_* = 161 kDa) especially when compared to preconsumer viscose, the extensibility of the concentrated cellulose species dominates and stabilizes the spinline.

**Table 2:** Fiber Spinning Data and Fiber Properties at a Conditioned Dry State

Samples	Spinning temperature (°C)	Draw Ratio (DR)	Elongation (%)	Tenacity (cN/tex)^a^	Linear density (dtex)^a^
Conditioned (dry)					
E5	42	10*^a^	7.2 ±1.3	36.3 ± 3.1	0.8 ± 0.04
E5B0.0005	50	10*^a^	3.4 ± 0.28	35.6 ± 1.28	0.7 ± 0.09
E5B0.0025	55	10*^a^	4.2 ± 0.73	33.5 ± 5.7	0.8 ± 0.15
V13	60	6	13.8 ± 1.4	21.1 ± 2.3	2.5 ± 0.3
		10*^a^	7.9 ± 1.3	23.9 ± 1.4	1.8 ± 0.04
V13B0.0065	70	6	14.4 ± 1.5	22.3 ± 1.5	2.4 ± 0.1
		10	7.9 ± 1.2	25.5 ± 1.9	1.7 ± 0.1
		12*^a^	8.1 ± 0.8	28.1 ± 1.8	1.4 ± 0.08

a Maximum draw ratio: 1 cN/tex = 10 N·m/kg and 1dtex = 10^–7^ kg/m in SI units.

By contrast, doping with HMWBC was found to improve the spinnability of the lower-molecular weight preconsumer viscose-based solution notably. The maximum draw ratio improved from 10 to 12, and the linear density of the resulting fiber consequently decreased from 1.7 dtex to 1.4 dtex (Table 2). Additionally, the optimum spinning temperature was higher.

SEM imaging was used to investigate the surface properties and diameter of the viscose waste-based fibers with, and without, the incorporation of HMWBC (Figure 5). All fibers have a round cross-section, smooth surfaces, and homogeneous morphology.^76^ The surface morphology of the fibers was not affected by doping with small amounts of HMW bacterial cellulose. However, the enhanced spinnability is reflected in the lower diameter of the fibers produced at the maximum draw ratio (Figure 5b,d). The fibers spun at a higher draw ratio are thinner (Figure 5c,d) due to the greater molecular stretching achieved and the reduced diameter of the liquid filament thread that can be stably achieved before entering the coagulation bath.

**Figure 5:**
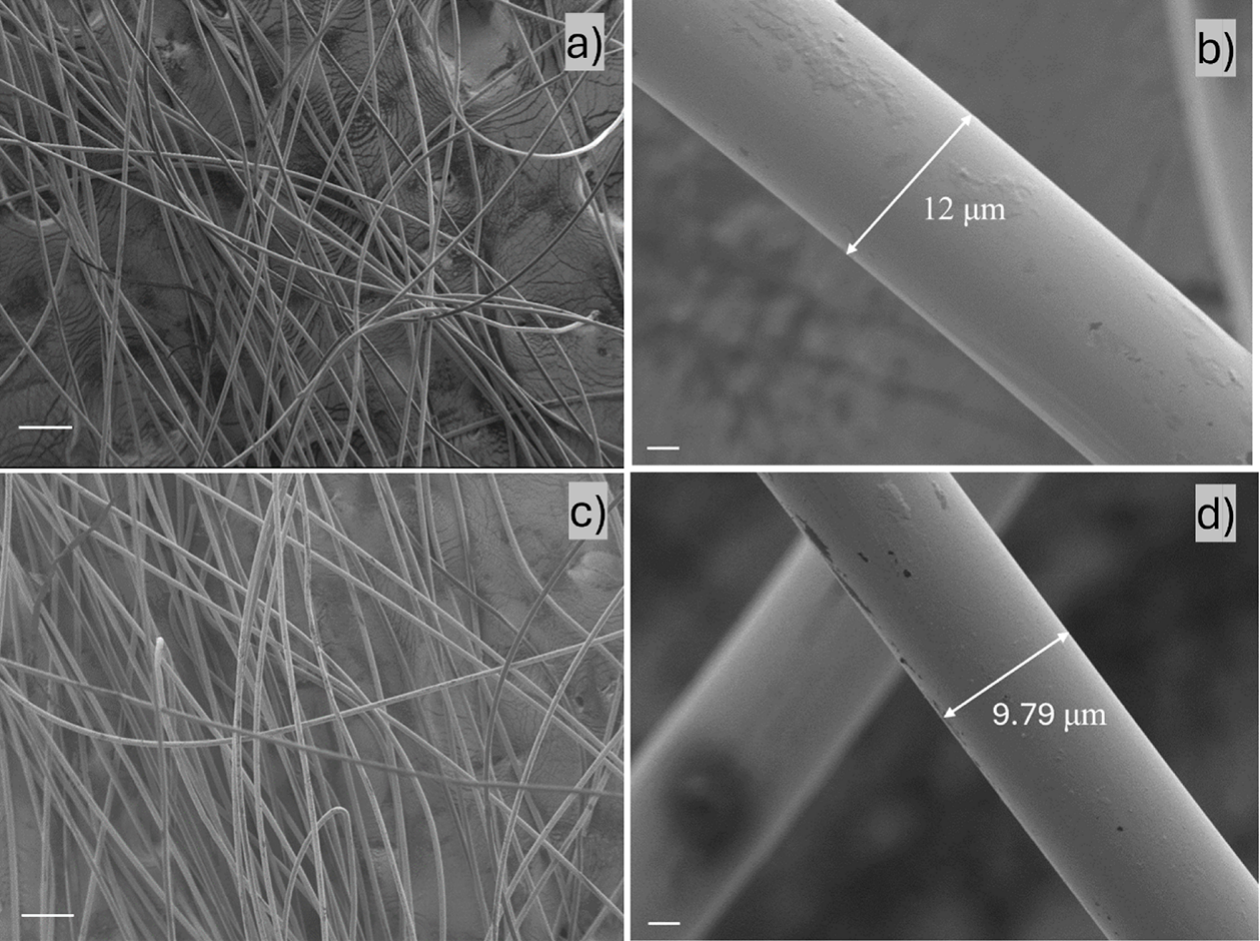
SEM images of spun fibers from 13 wt % preconsumer viscose-based cellulose (a and b) without HMWBC (Sample V13); (c and d) with HMWBC (sample V13.B0.0065). The scale bar on the left-hand side is 100 μm and on the right-hand side is 2 μm.

### Transient Extensional Rheology Measurements

Polymer melts and concentrated solutions exhibit pronounced nonlinear elastic effects in extensional flows due to the flow-induced orientation and stretching of macromolecules, with the level of the tensile stress difference governed by the principal stretch history.^77,78^ The transition from linear viscoelastic to nonlinear elastic behavior is influenced by the deformation rate, the total deformation attained (i.e., the Hencky strain), the concentration of the polymer and the relaxation time distribution of the material.^79^ In an elongational flow, the tensile stress difference Δ*σ* is an appropriate measure of the total stress in the system, since the principal axes of the stress and the deformation rate tensors are colinear. By contrast in a shear flow, the shear stress does not completely reflect the magnitude of the extra stress tensor due to the normal stress differences, causing the principal axes of stress and strain to differ.^80^ These differences will become important as we explore the transient extensional stress response of the different spinning dopes.

### Capillary Breakup Extensional Rheometry (CABER)

With the addition of HMWBC, the rate of filament thinning (Figures 6a and 7a), the transient extensional viscosity (Figures 6b and 7b), and the relaxation time (Table S3) of the doped solutions all increase. The extent of the increase in elongational viscosity is clearly evident for both 5 wt % PHK cellulose solution (Figure 6b) and the lower molecular weight 13 wt % preconsumer viscose-based cellulose solutions (Figure 7b). Adding only 0.0025 wt % of the high molecular weight bacterial cellulose (corresponding to 1 wt % of the total cellulose content) increased the relaxation time two-fold for the 5 wt % PHK cellulose-IL solutions (Table S3). Furthermore, the addition of a minute fraction of 0.0065 wt % of HMWBC (0.5 wt % of the total cellulose content) increased the relaxation time from λ = 39 s to λ = 49 s for the preconsumer viscose-based cellulose-IL solution (Table S3). This increase in relaxation time correlates directly with the increasing spinnability of the HMWBC-incorporated preconsumer viscose-based cellulose solution documented in Table 2. In conjunction with the increase in relaxation time, the doping of HMWBC into the spinning solutions clearly results in an increase in the apparent transient extensional viscosity at higher strains (Figures 6b and 7b). The transient extensional viscosity function, which is measured in CABER by the axial stretching of fluid elements under the action of capillary pressure, reflects how viscoelastic stresses in the air gap of the spinline will vary strain and deformation rate before the material enters the coagulation bath. The monotonically increasing nature of the transient extensional viscosity helps stabilize the spinline against perturbations, allowing for faster spinning rates and enabling the filament to be stretched to higher draw ratios and thinner fibers.

**Figure 6:**
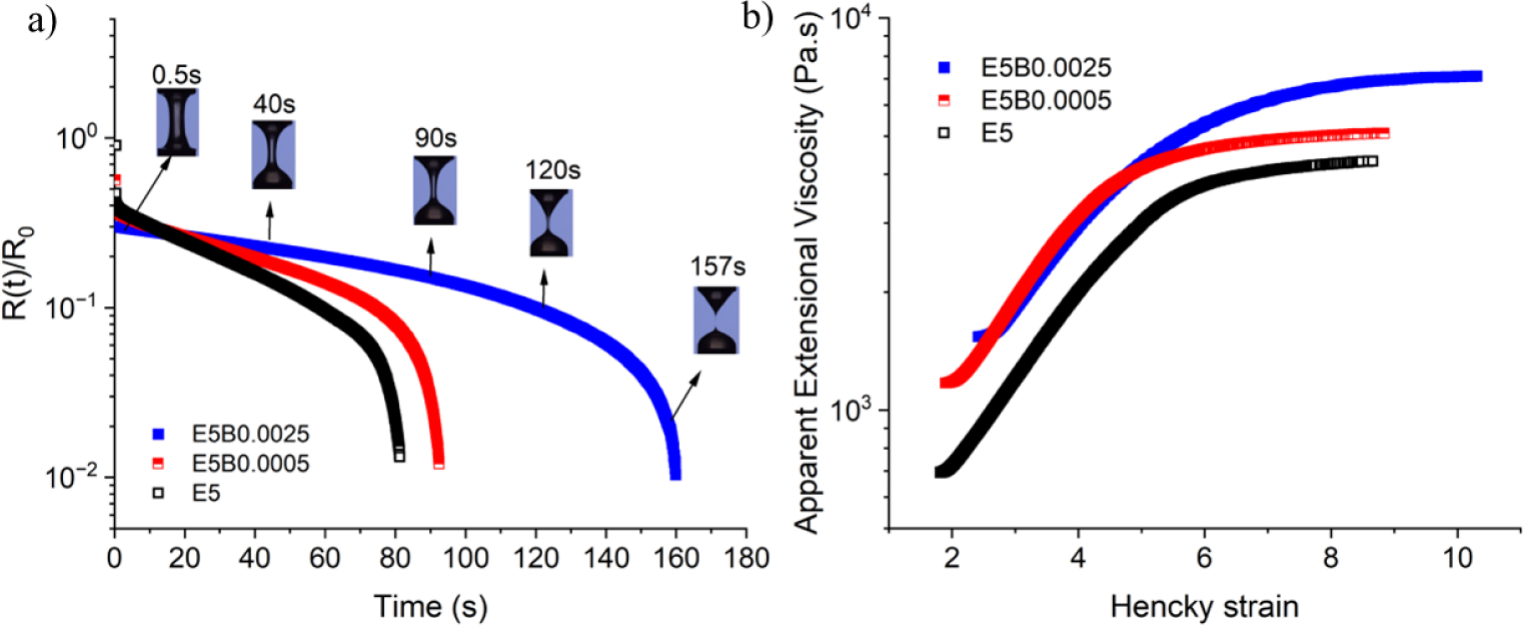
Capillarity-driven thinning of cellulose solutions; (a) filament profiles and rate of thinning for the 5 wt % PHK pulp solution with (red, blue symbols) and without (black squares) addition of HMWBC; (b) the corresponding evolution in the apparent extensional viscosity as a function of Hencky strain (maximum strain ∼ 10).

**Figure 7:**
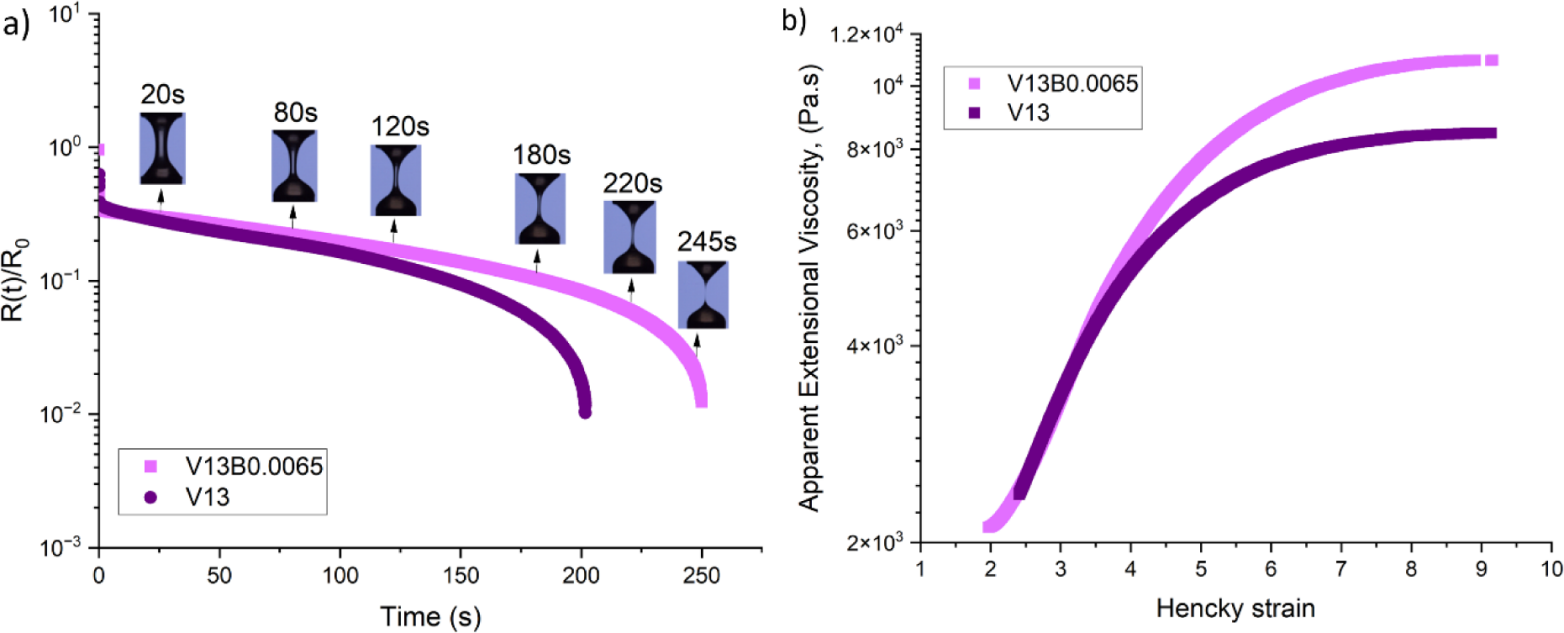
(a) Evolution of capillary-driven thinning profiles for the preconsumer viscose-based cellulose solution in [DBNH]­[OAc] with, and without, HMWBC; (b) corresponding values of the apparent extensional viscosities as a function of Hencky strain (maximum Hencky strain ∼ 9.3). The addition of 5% HMWBC increases the steady extensional viscosity achieved at high Hencky strains by 33%.

### Exponential Shear Rheology

Because access to capillary-thinning devices for measuring transient extensional rheology is limited (especially for polymer melts), we also explored the application of another strong flow, exponential shear,^49,51^ for assessing the stretchability of the different spinning dopes. In exponential shear rheometry, the instantaneous shear rate is increased exponentially (cf. eq 9) to mimic the kinematics of a steady planar extensional flow. Both steady homogeneous extension and exponential shear flow are examples of kinematically strong flows in which material points separate exponentially in time and thus generate a high level of molecular stretching.

One material function that was originally suggested^50^ as a proxy for the transient elongational viscosity in an exponential shear flow can be expressed in the form of the time-varying principal stress difference:

11 $$\eta_{p,ES}^{+}=\frac{\Delta \sigma^{+}\left(t\right)}{\alpha }=\frac{\sqrt{N_{1}^{2}\left(t\right)+4\sigma {\left(t\right)}^{2}}}{\alpha }$$

where, $\eta_{p,ES}^{+}$ is the stress growth material function measured in exponential shear (i.e., a measure of the transient elongational viscosity); *N*_1_ is the first normal stress difference generated in the strongly sheared viscoelastic fluid; σ is the corresponding shear stress and α is the deformation rate (in s^–1^). It is noted that this material function has been shown to be an effective measure of the transient extensional viscosity specifically in cases of high Weissenberg number (*Wi* ≫ 1),^81^ where the Weissenberg number is a dimensionless measure of flow strength defined as the product of the strain rate and the fluid relaxation time: *Wi* = αλ (using the relaxation times given in Table S3). We note that other choices of an appropriate extensional stress growth material function may also be useful and informative for other material systems, and we guide readers to further in-depth discussions on this material function definition elsewhere.^52,81^

In the present study, the samples used for exponential shear span approximately the range of *Wi* = 157.5 to 345.8, and are thus certainly in the *Wi* ≫ 1 limit. Consistent with this limit, we can use the material function defined in eq 11 to calculate a quantitative metric for assessing the transient planar extensional viscosity of the different spinning dopes. An example is shown in Figure 8 for the E5 reference sample. For all exponential deformation rates examined (α = 1–7 s^–1^) the Weissenberg number is well above unity (*Wi* = 22.5–157.5), indicating that molecular stretching dominates the extensional flow response. Consequently, while the time-varying principal stress difference Δσ^+^(*t*) varies significantly with α (Figure 8a), the curves of the corresponding material function $\eta_{ES}^{+}$ collapse onto similar trajectories when plotted against Hencky strain α*t* (Figure 8b). This self-similarity confirms that, in the high-*Wi* limit, the transient extensional response is primarily a function of accumulated strain rather than the absolute value of α. Therefore, in our subsequent comparisons investigating the key role of cellulose concentration and the HMW bacterial cellulose additive, we choose to compare results at only one representative deformation rate (α = 7 s^–1^) for clarity.

**Figure 8:**
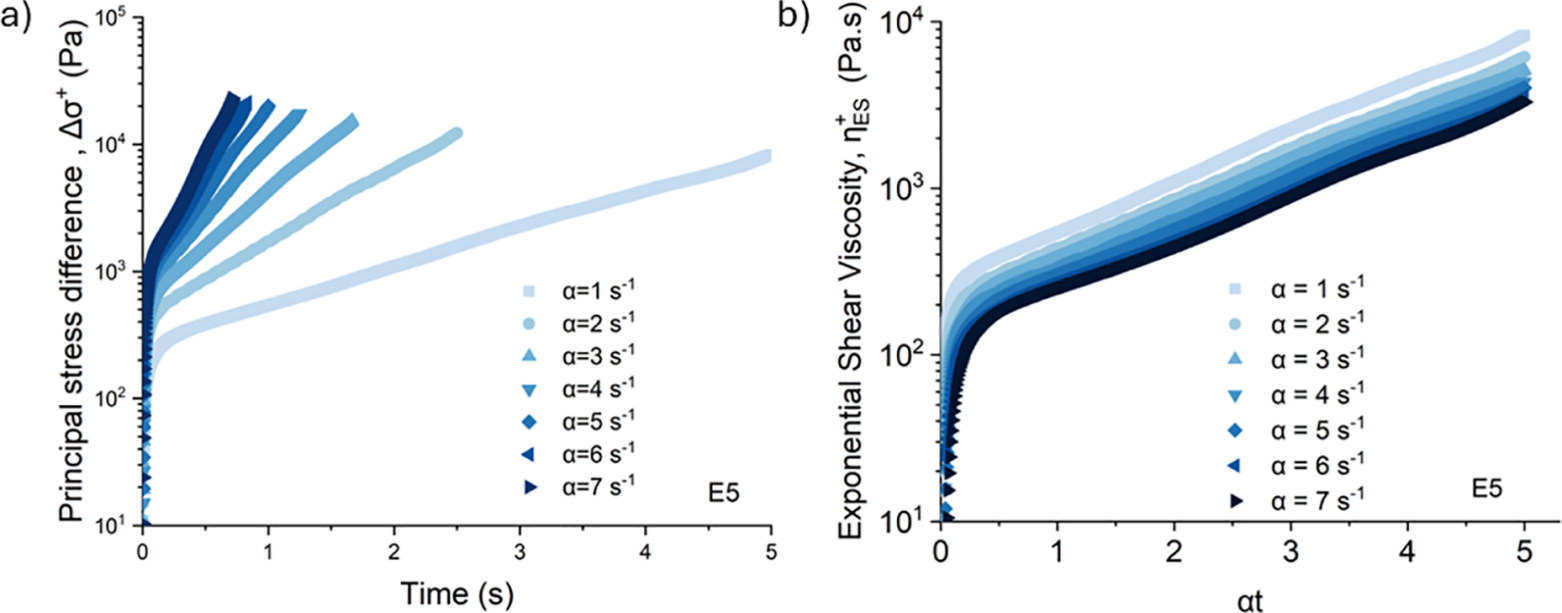
Comparison of exponential shear flow data for sample E5 (at 60 °C) at deformation rates α = 1–7 s^–1^, corresponding to Weissenberg numbers *Wi* ≫ 1 (*Wi* = 22.5–157.5). (a) Evolution of the principal stress difference Δσ^+^ vs time shows a strong dependence on the imposed deformation rate. (b) When plotted as a transient extensional viscosity $\eta_{ES}^{+}$ vs Hencky strain ε = αt, the curves collapse onto similar trajectories, indicating that in the high-Weissenberg number limit the transient strain-stiffening material response is dominated by accumulated strain rather than the absolute value of α.

The purpose of these exponential shear flow tests is to compare the transient extensional properties of different cellulose compositions and demonstrate the key role of doping with high molecular weight bacterial cellulose at realistic temperatures and stretching rates that emulate spinning conditions. We first investigate changes in the exponential shear rheology with concentration, as shown in Figure 9. For each sample, the first normal stress difference and shear stress were measured as a function of time at α = 7 s^–1^. These two experimental measurements can each be plotted individually against Hencky strain (as shown in Supplementary Figures S9–S15). We note that at high strain amplitude, the total principal stress is dominated by the first normal stress difference, an indication of strong nonlinear viscoelastic stress contributions. The total principal stress in the direction of stretching (Δσ^+^(t)) is computed using eq 11 and averaged over three independent experiments for each material composition and temperature. Each set of three runs was performed to confirm that the standard deviation is small and that sample loading imperfections (which can be amplified exponentially during the test) are minimal between repeated experiments. For each material, we use this total principal stress to compute a measure of the transient extensional viscosity, by normalizing by the effective Hencky strain rate, α (cf. eq 11). The processed data for all these experiments is shown in Figures 9 and 10, to illustrate the key role of concentration and molecular composition on the principal stress growth in a strong elongational flow. As shown in Figure 9, increasing the concentration of dissolved cellulose increases the tensile stress in the spinline especially at high Hencky strains (which are relevant to the processing conditions of fiber spinning).

**Figure 9:**
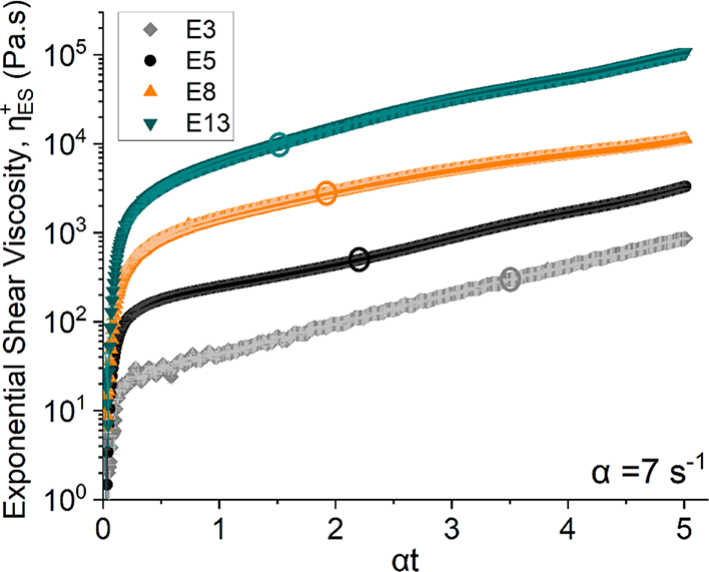
Transient extensional rheology of different concentration spinning dopes determined by exponential shear: the exponential shear viscosity of different concentrations (3, 5, 8, 13 wt %) of PHK pulp solution in [DBNH]­[OAc] as a function of the accumulated Hencky strain ε = αt for α = 7 s^–1^. Number of reported experiments *n* = 3 for each composition. The measurement temperature for all of the dopes was 60 °C, except for highly viscous 13 wt % solution which is processed at 80 °C. The large hollow circle defines the Hencky strain beyond which the total principal stress is dominated by the first normal stress difference, indicating that the elastic normal stress difference dominates.

**Figure 10:**
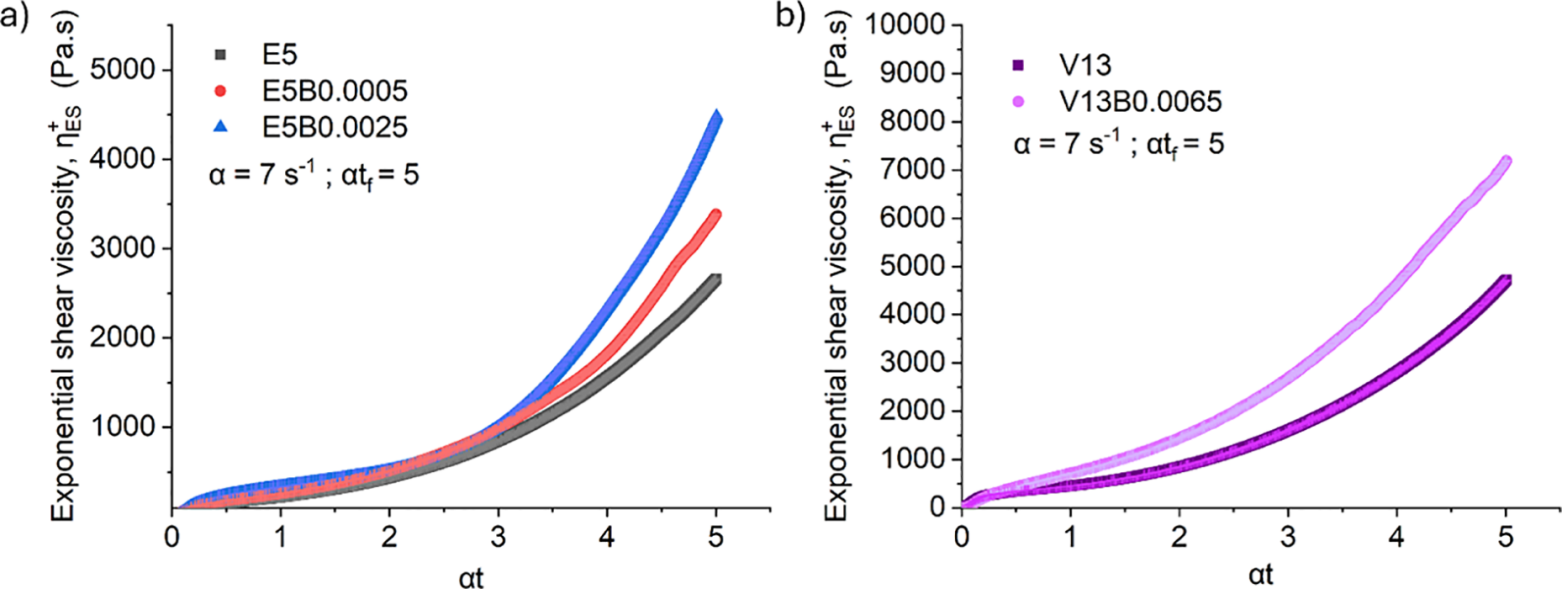
Transient extensional rheology probed by exponential shear: (a) the exponential shear viscosity of (a) 5 wt % PHK pulp solution (b) 13% viscose waste-based solution (V13) in [DBNH]­[OAc] with and without HMWBC as a function of the accumulated Hencky strain ε = αt for α = 7 s^–1^.

Remarkably, even trace additions of the HMWBC substantially enhanced the extensional response of the spinning dopes. For the 5 wt % PHK pulp solutions shown in Figure 10a, as little as 0.0005 wt % HMWBC (i.e., 1% of the total polymer present) increased the exponential viscosity by ∼30% (at a Hencky strain of α*t* = 5), while a modest increase to 0.0025 wt % (5% of total polymer composition) yielded a ∼70% rise in the extensional viscosity. A similarly pronounced effect is observed for the preconsumer viscose-based solutions (Figure 10b), where the incorporation of just 0.0065 wt % HMWBC (0.5% of total polymer added) elevates the extensional viscosity by up to 50% at high Hencky strains. These substantial enhancements, achieved at ultralow polymer loadings, are reflected in the increased values of the extensibility-averaged molecular weight *M_L_* (cf. Table 1) and—as we document below—directly correlate with the improved spinnability of the doped cellulose solutions.

Previous studies^32^ have shown that the extensibility-averaged molecular weight more appropriately captures the influence of a small fraction of long, highly extensible chains on extension-dominated processes such as electrospinning or fiber spinning. In such bidisperse or doped blends, the contribution of these long chains to the total tensile stress difference is amplified by their large chain extensibility, leading to the disproportionately large impact on the total extensional viscosity of the spinning solution we have just documented.^32,36^ To quantify these effects, we have defined a single concentration-dependent measure of the extensibility-averaged molecular weight $cM_{L}^{1+\nu }$ (Table 1) which should provide a direct measure of how molecular extensibility controls the extensional viscosity and the subsequent spinnability of the different dopes. As we show in Figure 11, both the limiting zero-shear-rate viscosity (η_0_) and the maximum value of the exponential shear viscosity $\eta_{ES}^{+}$ (reported at a constant final Hencky strain ε*_f_* ≡ α*t_f_* = 5) follow power-law dependences on $cM_{L}^{1+\nu }$ , with excellent fits (*R* ^2^ = 0.97). The scaling exponents are 2.65 for η_0_ and 3.05 for $\eta_{ES}^{+}$ , respectively, indicating that the extensional viscosity increases more rapidly than the zero-shear viscosity with increasing values of $cM_{L}^{1+\nu }$ . The increasing vertical separation between the two curves illustrates the greater sensitivity of extensional flows to the chain extensibility. Notably, even trace additions of HMWBC additive (e.g., E5B0.0005 (semifilled squares), E5B0.0025 (filled squares), V13B0.0065 (filled triangles)) substantially increase the extensional viscosity, highlighting the significant impact of low concentrations of high molecular weight additives on the nonlinear rheological properties relevant to spinning operations.

**Figure 11:**
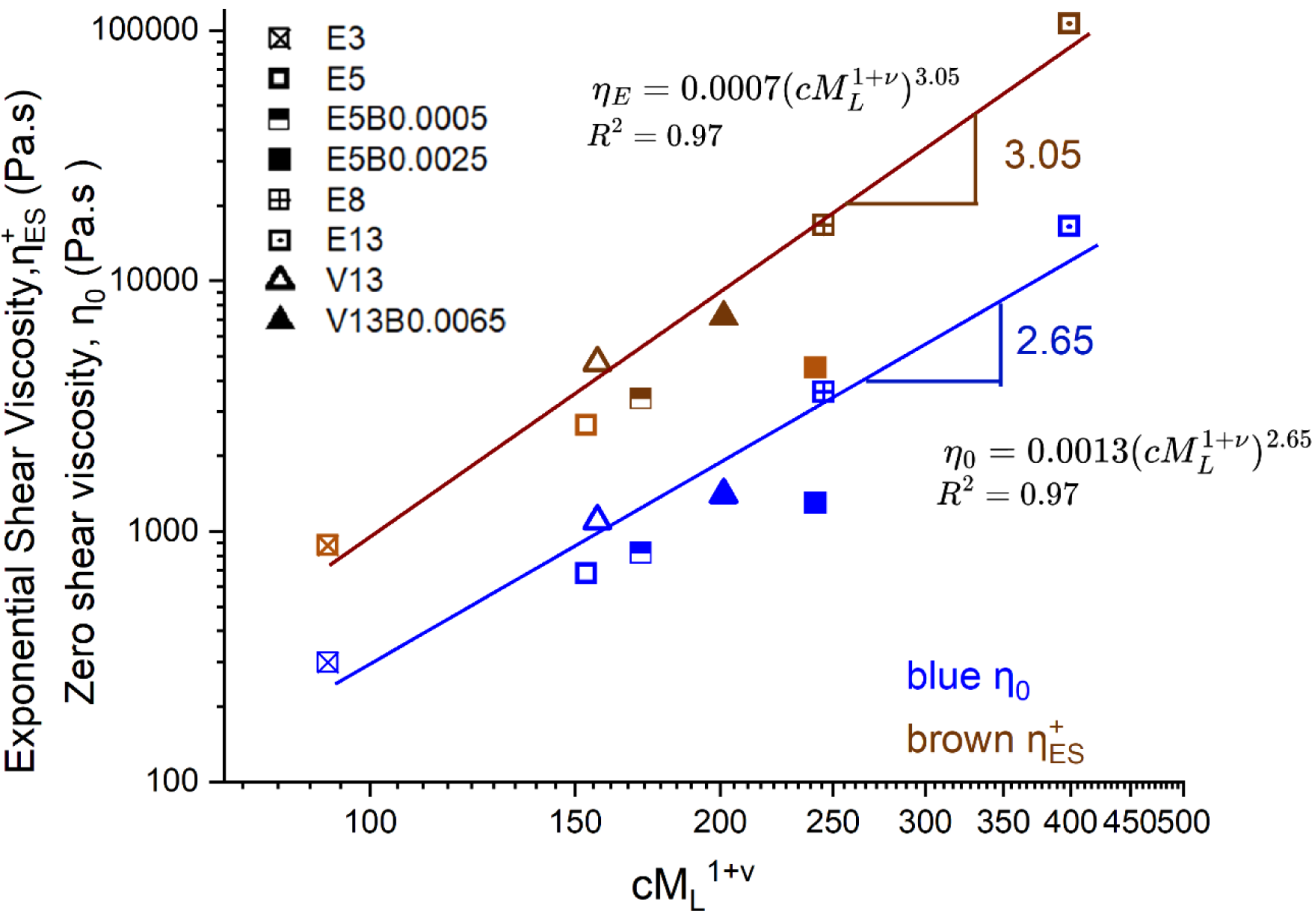
Evolution in the apparent extensional viscosity $\eta_{ES}^{+}$ (brown symbols) and zero shear viscosity η_0_ (blue symbols) as a function of $cM_{L}^{1+\nu }$ .

For non-Newtonian fluids, the ratio of the extensional viscosity to shear viscosity is commonly defined as the Trouton ratio, *Tr* = η*_E_*/η_0_.^82^ This ratio is identically equal to *Tr* = 3 for a Newtonian fluid in uniaxial extension; however, the value can vary widely for dilute and concentrated polymer solutions depending on the degree of chain overlap and molecular extensibility.^65,79^ As the imposed deformation rate increases, the extensional viscosity typically evolves at a different rate compared to the (decreasing) shear viscosity, leading to higher values of the Trouton ratio.^83,84^ In Figure 12 we plot the maximum draw ratio obtained in the spinning experiments for each polymer solution (taken from Table 2) against the value of the Trouton ratio calculated from the steady shear rheology and the exponential shear rheology (at the final Hencky strain of ε*_f_* = 5). As the extensional viscosity (and Trouton ratio of the spinning dopes) is increased—either through addition of trace amounts of HMWBC (e.g., sample V13B0.0065), or through increasing the degree of entanglement and shear-thinning (samples E8 and E13)—the maximum draw ratio that can be stably obtained in the fiber-spinning operation increases and the minimum fiber diameter concomitantly decreases.

**Figure 12:**
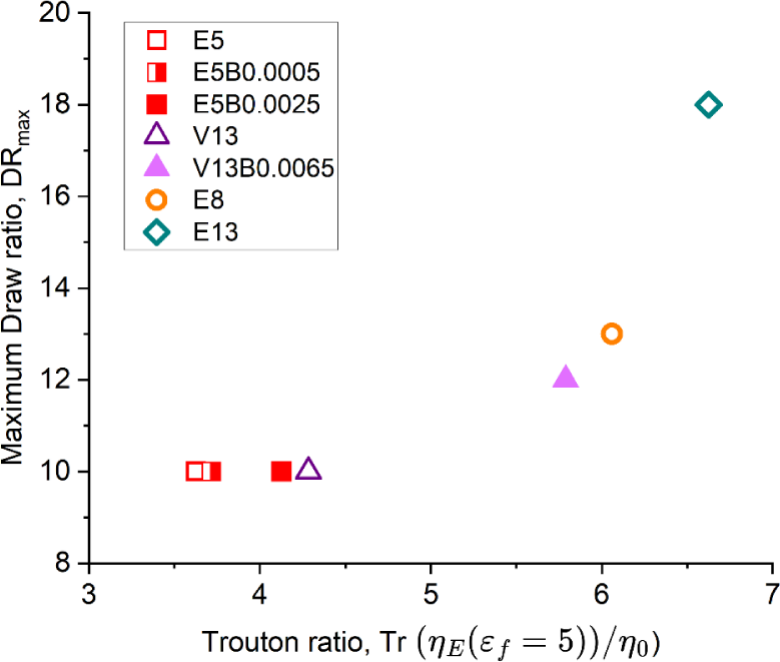
Maximum draw ratio attainable as a function of the Trouton ratio calculated from shear viscosity, η_0_ , and exponential shear rheology at high strain rate (*Wi* ≫ 1) and a final Hencky strain ε_*f*_ = 5.

These findings are in good qualitative agreement with the capillary-thinning experiments discussed above (cf. Figures 6 and 7). An increasing trend in the measured Trouton ratio is also observed in the CaBER tests for the HMWBC-incorporated preconsumer viscose-based solution that are associated with higher values of spinnability (Table S3). Of course the specific values of the extensional viscosities are quantitatively different for CaBER and exponential shear rheology. This is not unexpected, as CaBER involves slow capillarity-driven uniaxial stretching of an axisymmetric filament (where the elastic and capillary forces balance and set an effective stretching rate, ε̇ = 2/3λ),^44^ while exponential shear rheology involves transient planar kinematics imposed by a controlled deformation rate in the bulk (which we set to ε̇ = 7 s^–1^).

Despite these important distinctions in the two sets of experiments, both sets of transient extensional rheological measurements show similar trends of increasing Trouton ratios at higher strains, which correlate strongly with the enhanced spinnability of the doped solutions and the corresponding changes in the viscoelastic properties. These results suggest that exponential shear rheology can be an effective and relatively accessible test protocol for determining the spinnability of different cellulosic solutions and investigating the influence of different factors such as concentration, presence of additives, and variations in polymer chain length. Our measurements shown in Figures 11 and 12 suggest that doping of high molecular weight bacterial cellulose to increase the extensibility-averaged molecular weight is an effective method to increase the spinnability of low molecular weight-grade cellulose solutions.

### Life Cycle Assessment and Sustainability Considerations

Several life-cycle assessment (LCA) studies have already examined the environmental impacts of both bacterial cellulose production and cellulose-based textile spinning routes. Silva et al. conducted a detailed “cradle-to-gate” LCA of bacterial cellulose pulp and BC-Lyocell fibers, showing that the dominant environmental contributors arise from culture medium preparation, washing, and energy use, while also identifying clear pathways for impact reduction through process optimization and byproduct utilization.^85^ Importantly, this work demonstrated that bacterial cellulose can be assessed independently of the other cellulose components (because its production route is so distinct) and that its environmental footprint is highly sensitive to fermentation and downstream processing assumptions. In parallel, life cycle analyses of the Ioncell process have shown that solvent-based cellulose fiber spinning routes using recyclable, nontoxic solvents can achieve both (i) favorable environmental performance (relative to the viscose process), and also (ii) competitive performance relative to the NMMO-based Lyocell process (particularly with respect to water use and chemical toxicity).^86^ Finally, qualitative and screening-level LCAs for emerging textile technologies consistently emphasize that, at low additive or dopant loadings, the overall environmental impact of regenerated cellulose fibers is dominated by the bulk cellulose feedstock and spinning process rather than by those of minor functional additives.^87^ Together, these studies indicate that, at the ultralow loadings (*c* < c*) used here, the incremental contribution of bacterial cellulose to total life-cycle impact is expected to be secondary, and that a meaningful LCA of the incremental costs associated with HMW bacterial cellulose incorporation into spinning dopes would first require a fully defined industrial process scenario.

## Conclusions

Developing new strategies for recycling cellulose-based textiles is crucial for a sustainable environment. The present work shows that doping a semidilute or concentrated cellulose solution with a very small fraction (less than the critical overlap concentration c*) of high molecular weight bacterial cellulose (HMWBC) can significantly improve the spinnability of viscose-based textile waste, which is characterized as a low molecular weight cellulose substrate. Additionally, we have performed an extensive set of rheological measurements to understand the systematic changes in the rheology of a family of cellulose-[DBNH]­[OAc] solutions, considering changes in temperature, concentration, and the incorporation of trace amounts of the HMWBC dopants. These tests include small amplitude oscillatory shear sweeps, capillary-driven thinning measurements of the transient extensional rheology, and exponential shear tests to probe a strong transient extensional flow at realistic deformation conditions and temperatures, representative of fiber spinning operations.

Using the principles of time–temperature and time–concentration superposition, we have shown how master curves for the rheological properties of the cellulose matrix dissolved in [DBNH]­[OAc] can incorporate the observed variations with temperature, concentration, and addition of dopant. CaBER measurements showed that the higher spinnability and narrower-diameter fibers observed experimentally directly correlate with longer relaxation times and higher values of the transient extensional viscosity. We have also quantified the additional extensional stress generated by adding a very small fraction of bacterial cellulose to a spinning dope using exponential shear rheology.

We have introduced and evaluated an extensibility-averaged molecular weight *M_L_* which better captures the response of a polydisperse macromolecular system to large extensional deformation. Since we compare a large number of samples with a range of compositions prepared at varying concentrations, we show that the most representative parameter to evaluate and intercompare formulations is the concentration-dependent extensibility-averaged molecular weight, $cM_{L}^{1+\nu }$ . Increasing the value of $cM_{L}^{1+\nu }$ for preconsumer textile waste-based solutions (which are typically characterized by low values of their viscosity-averaged molecular weight, *M_V_*) via addition of a small fraction of high molecular weight bacterial cellulose (HMWBC) provides a significant increase to the extensional viscosity. This enhancement is substantially greater than the impact on the zero-shear viscosity, and we find that the resulting increase in the Trouton ratio *Tr* = η*_E_*/η_0_ correlates very well with the enhanced spinnability of the doped cellulose solutions that is observed in dry-jet wet spinning. The enhanced spinnability enables thinner fibers (lower denier) to be obtained. As shown in Table 2, the higher draw ratio also leads to small enhancements in the tensile strength (tenacity) and elongation of the spun fibers. There is negligible change in the fiber morphology obtained because the amount of high-molecular-weight bacterial cellulose present in the final dry fiber is small.

This study has the potential to significantly influence industrial perspectives of textile recycling. The approach is cost-effective because (i) we use existing raw material sources, including low-DP textile waste streams and naturally derived bacterial cellulose, without additional processing steps, and (ii) the mass fraction of the high molecular dopant that needs to be added is very small (below c*). This rational approach can potentially be extended to other textile waste streams (e.g., postconsumer textile waste with even lower viscosity-averaged molecular weights). As we have demonstrated in this work, probing the state of stress that develops in spinning dopes using different rheological test methodologies provides a powerful means to evaluate the spinnability of specific material formulations and cellulosic blends early in the processing sequence. We have explored two transient extensional rheometry techniques that offer complementary insights into measuring and controlling the extensibility of polydisperse macromolecular solutions: (1) capillarity-driven thinning (in a CaBER device), directly probes the rate of filament thinning dynamics under the action of surface tension and the time (or strain accumulated) to breakup; (2) exponential shear rheology, which can be performed on conventional shear rheometers, assesses the transient extensional response of a spinning dope at large strain rates and high Hencky strain. These techniques can also be applied to quantify other extension-dominated processing operations beyond fiber spinning. We believe the rheological understandings provided by this study can guide future developments and optimization of lyocell-based spinning processes.

Future work will extend this rheology-guided framework to alternative high-molecular-weight cellulose sources to evaluate scalability and cost-effectiveness. While the present study is deliberately focused on establishing structure–rheology–spinnability relationships, comprehensive life-cycle and technoeconomic analyses of bacterial cellulose production and incorporation will be essential for assessing industrial viability. Such analyses are best addressed once specific fermentation, purification, and processing routes are defined and optimized.

## Supplementary Material



## Notes

Raw material characterization data (intrinsic viscosity, molar mass distribution, degree of polymerization) (Table S1); sample compositions and dopant concentrations in [DBNH]­[OAc] (Table S2); spinning parameters and rheological metrics, including zero-shear viscosity, maximum draw ratio, capillary breakup time, relaxation time, and Trouton ratio (Table S3); SAOS time–temperature superposition master curves (Figures S1–S8); concentration- and dopant-dependent viscosity trends (Figure S7); polymer stress difference, normal stress, and shear stress data for all formulations (Figures S9–S15) (PDF)
