# Supplementary material for: Enhancing the Spinnability of Cellulose-Based Textile Waste by Doping with High Molecular Weight Bacterial Cellulose
Source: Biomacromolecules. 2026 Mar 6;27(4):2612–28. doi: 10.1021/acs.biomac.5c02370 (PMC13080776; doi:10.1021/acs.biomac.5c02370)
Supplement: Supplementary file 1 [file bm5c02370_si_001.pdf]

Enhancing the spinnability of cellulose-based textile waste by doping with high molecular weight bacterial cellulose.

Kaniz Moriam[1,2], Crystal E Owens[1,3], Laurel Kroo[1], William Ghann [4,] Jamal Uddin[4], Leena Pitkänen[2], Michael Hummel[2*], Gareth H McKinley[1*]

[1]Hatsopoulos Microfluids Laboratory, Department of Mechanical Engineering, Massachusetts Institute of Technology, Cambridge, MA 02139, USA

[2]Department of Bioproducts and Biosystems, Aalto University, Espoo, Finland

[3]Computer Science and Artificial Intelligence Laboratory, Massachusetts Institute of Technology, Cambridge, MA 02139, USA

[4] Center for Nanotechnology, Department of Natural Sciences, Coppin State University, Baltimore, Maryland, MD 21216

**Corresponding authors*

Date: 11.02.2026

Raw material characterization

Table S1: Intrinsic viscosity and molar mass distribution measurement results. The value of ν was calculated from the relation, $\nu = \frac{a+1}{3}$

	Intrinsic viscosity data					Size exclusion chromatography data				
	Intrinsic viscosity η (mLg^{-1})	a	ν	DP	M_v (kDa)	M_w (kDa)	M_n (kDa)	PDI	DP< 100 (%)	DP> 2000 (%)
Prehydrolysis kraft pulp (Enocell)	434 ±1	0.76	0.58	998	161	141	50	2.8	6.4	8.0
Pre-consumer Viscose waste	176 ±9	1	0.67	420	70	45	27	1.7	13	0
Bacterial cellulose	1456 ±52	0.76	0.58	4910	795	498	175	2.8	1.1	65

Table S2: Overview of the samples based on the concentration of the cellulose and amount of additive (bacterial cellulose).

Raw materials	Total conc. in ionic liquid [DBNH][OAc]	Additive conc. Based on cellulose (wt%)	Additive conc. In ionic liquid (wt%)	Sample name
Prehydrolysis kraft pulp (Enocell)	3	0	0	E3
	5	0	0	E5
	5	1	0.0005	E5B0.005
	5	5	0.0025	E5B0.0025
	8	0	0	E8
	13	0	0	E13
Pre-consumer viscose waste	13	0	0	V13
	13	5	0.0065	V13B0.0065

Table S3: The complex viscosity at spinning temperature from shear rheology; capillary break-up time, relaxation time, and Trouton ratio from CaBER analysis.

		**Spinning parameters**				**Rheological analysis**	
Samples	Spinning Temperature (˚C)	η_0 (at spinning temperatures) Pa.s	Maximum Draw ratio (DR_{max})	*ΔH* (at 50˚C) kJ/mol	Breaking time (s)	Relaxation time (λ) (s)	Trouton ratio (CaBER)
E5	45	680	10	60.1	81	22.5 ± 0.9	5.88
E5B0.0005	50	820	10	69.5	93	28.4 ± 0.6	5.60
E5B0.0025	55	1300	10	113.6	160	41.6 ± 0.2	7.97
E8	70	3600	13	67.1			
E13	75	16500	18	74.5			
V13	60	1100	10	95.1	200	39.5 ± 0.8	6.36
V13B0.0065	70	1400	12	100.6	250	49.4 ± 0.4	7.12

Time-temperature superposition from Linear Viscoelastic measurement

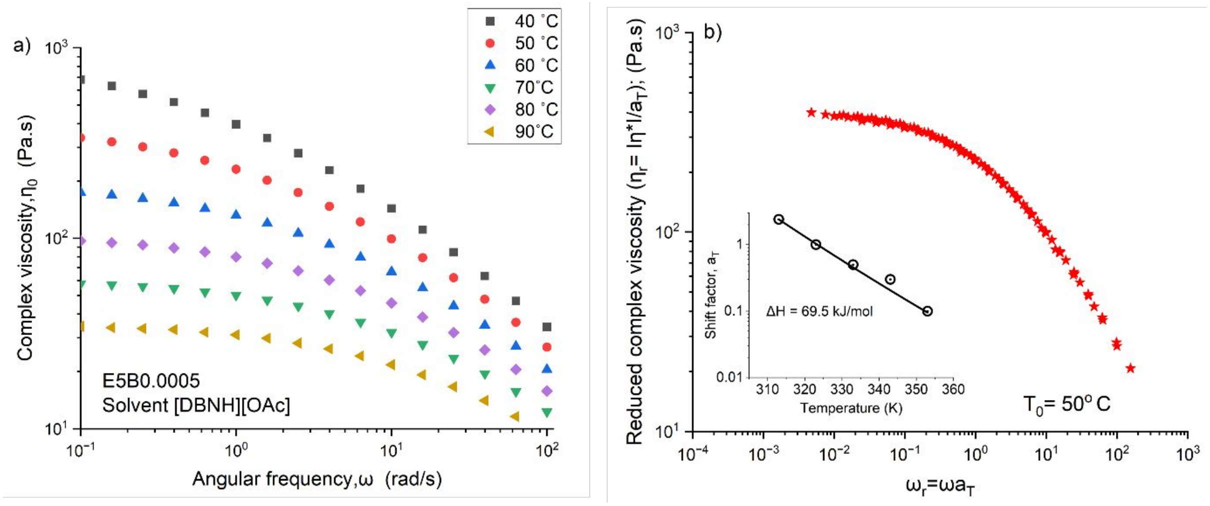

Figure S1. a) Complex viscosity as a function of angular frequency at different temperatures (range 40 to 80 °C) for sample E5B0.0005, b) the SAOS master curve via tTS referenced at 50 °C.

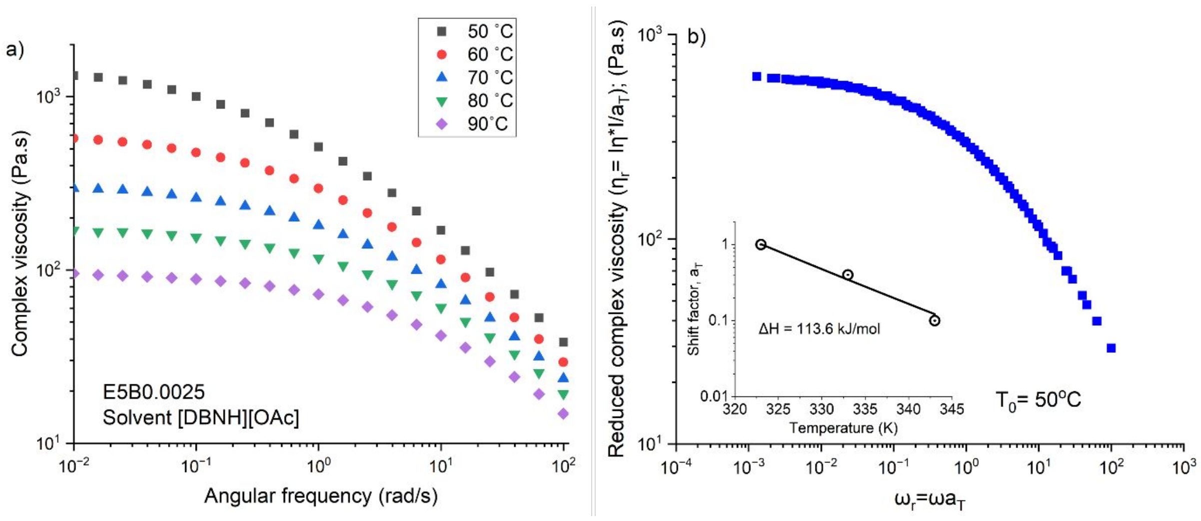

Figure S2. a) Complex viscosity as a function of angular frequency at different temperatures (range 40 to 80 °C) for sample E5BO.0025, b) the SAOS master curve via tTS referenced at 50 °C.

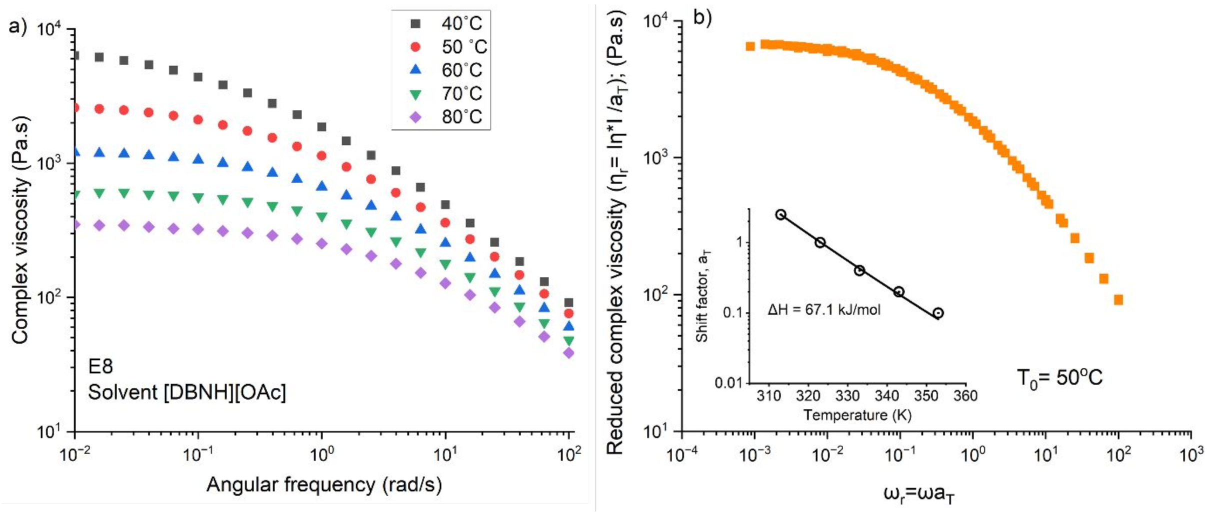

Figure S3; a) Complex viscosity as a function of angular frequency at different temperatures (range 40 to 80 °C) for E8 b) the SAOS master curve via tTS referenced at 50 °C.

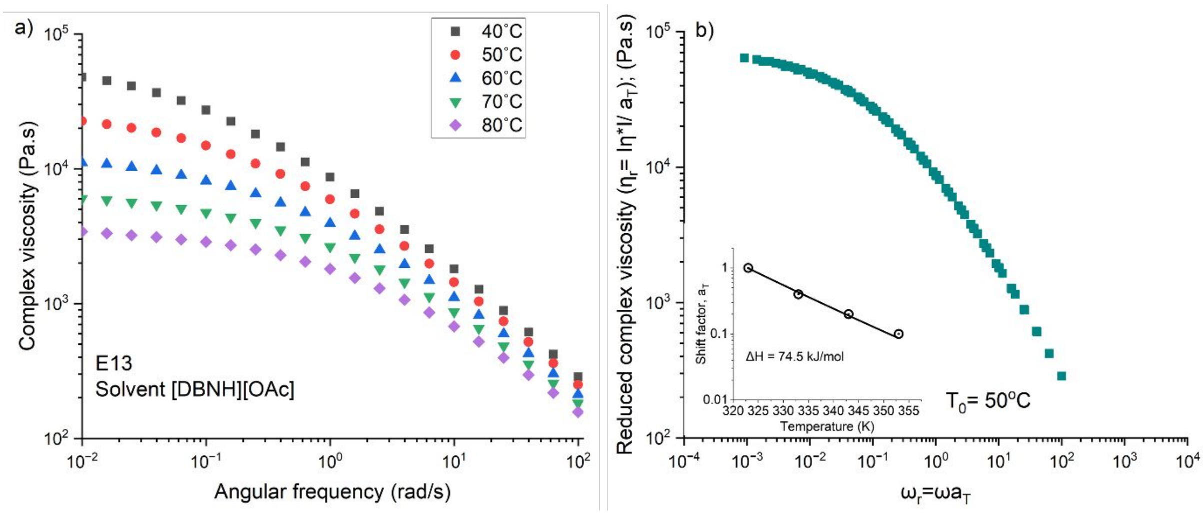

Figure S4. a) Complex viscosity as a function of angular frequency at different temperatures (range 40 to 80 °C) for E13, b) the SAOS master curve via tTS referenced at 50 °C;

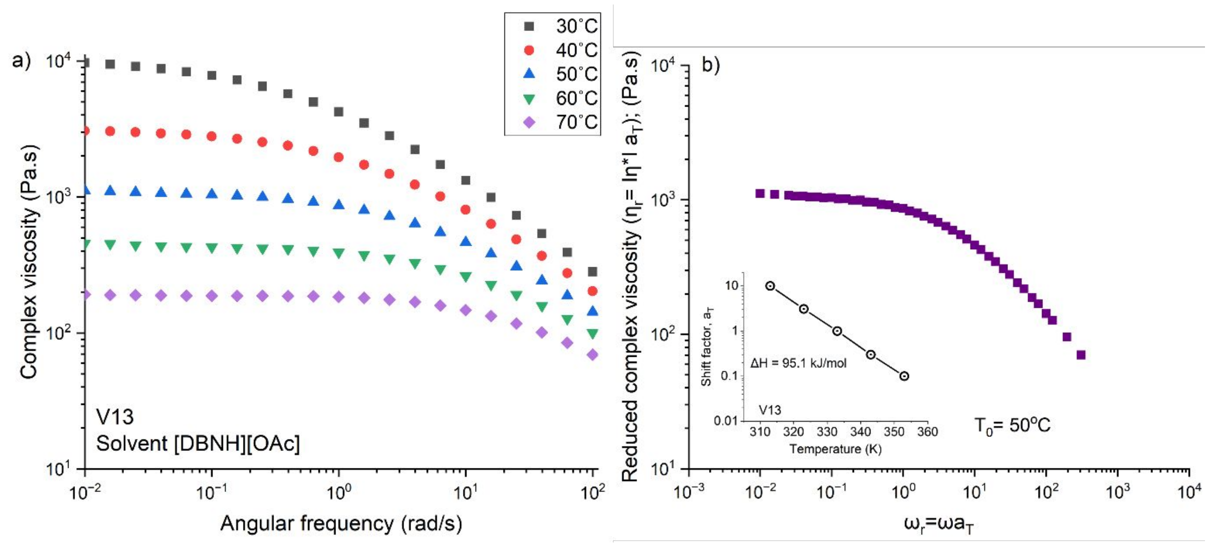

Figure S5. a) Complex viscosity as a function of angular frequency at different temperatures (range 30 to 80 °C) for V13, b) the SAOS master curve via tTS referenced at 50 °C.

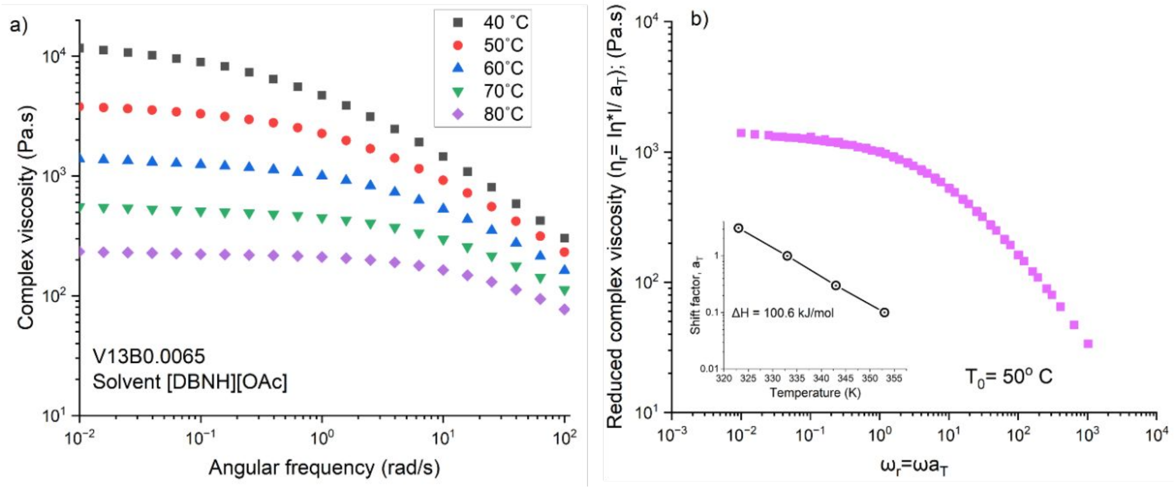

Figure S6. a) Complex viscosity as a function of angular frequency at different temperatures (range 40 to 80 °C) for V13B0.0065, b) the SAOS master curve via tTS referenced at 50 °C.

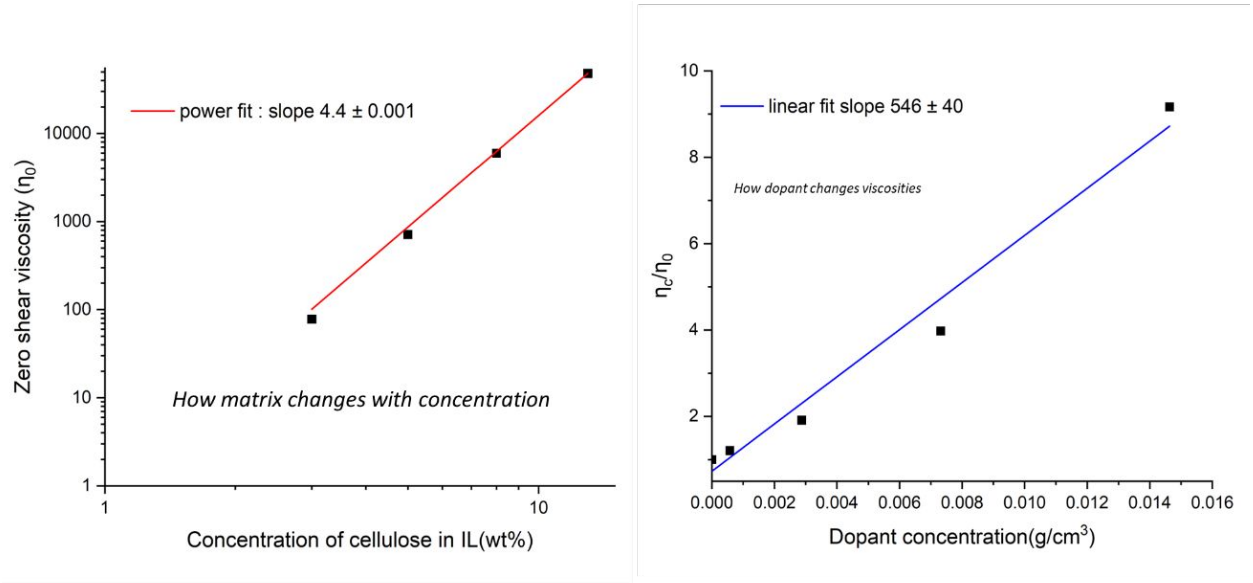

Figure S7: a) Zero shear viscosity as a function of concentration, b) relative viscosity as a function of the amount of dopant HMWBC in the cellulose matrix.

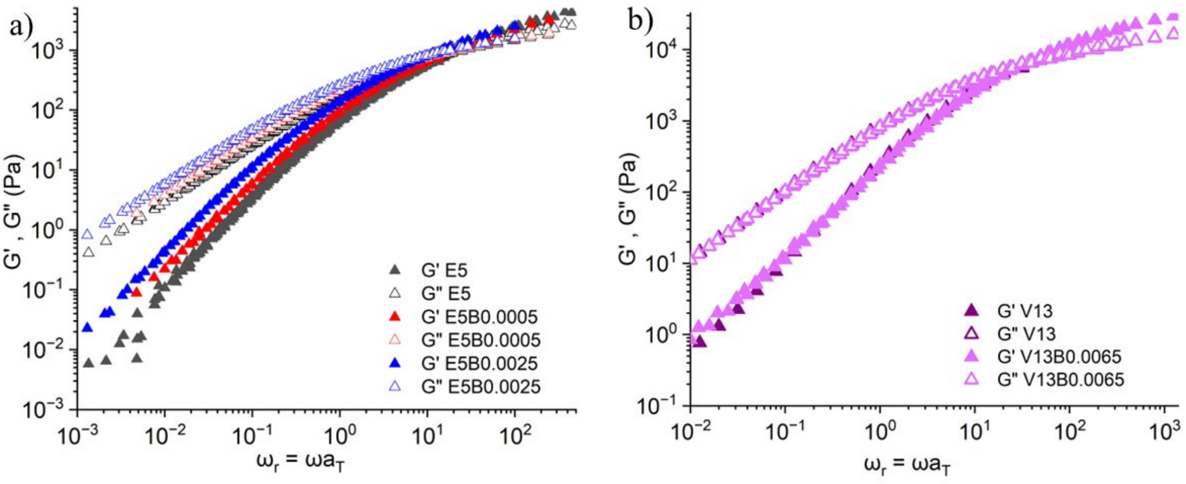

Figure S8: SAOS master curves showing the magnitude of dynamic modulus (as a function of frequency for a) PHK pulp solution in IL with, and without, HMWBC constructed via tTS at the spinning temperature of 50 °C, b) pre-consumer viscose based cellulose-IL solution with and without HMWBC

Polymer Stress Difference (PSD)/Normal stress/shear stress

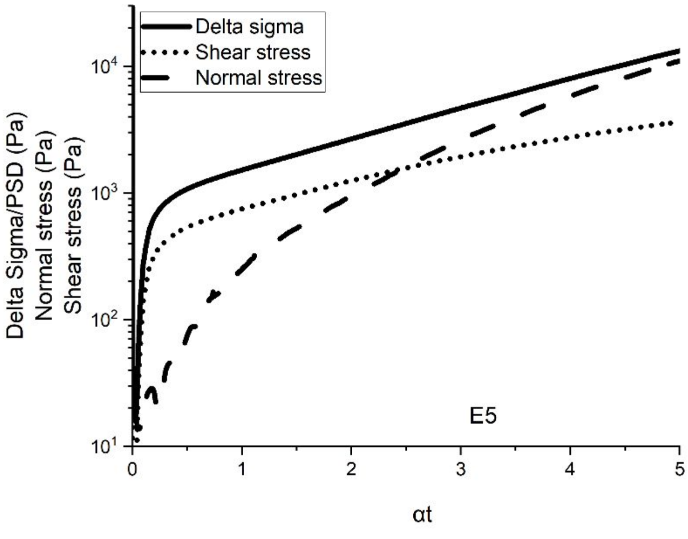

Figure S9: Polymer stress difference, normal stress, and shear stress for the sample E5.

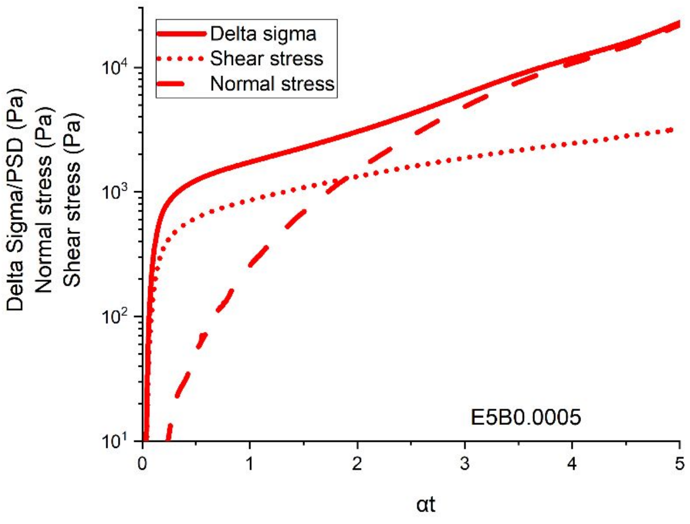

Figure S10: Polymer stress difference, normal stress, and shear stress for the sample E5B0.0005.

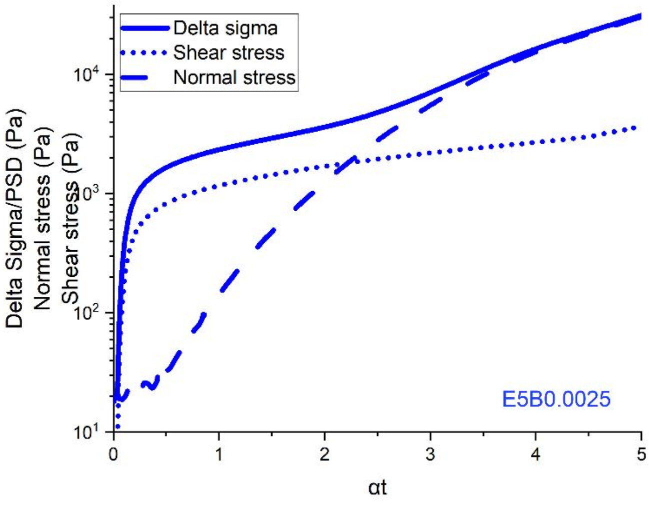

Figure S11: Polymer stress difference, normal stress, and shear stress for the sample E5B0.0025.

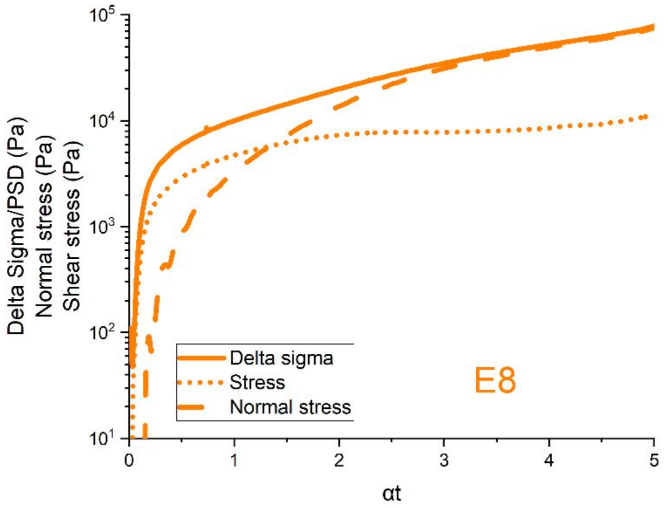

Figure S12: Polymer stress difference, normal stress, and shear stress for the sample E8.

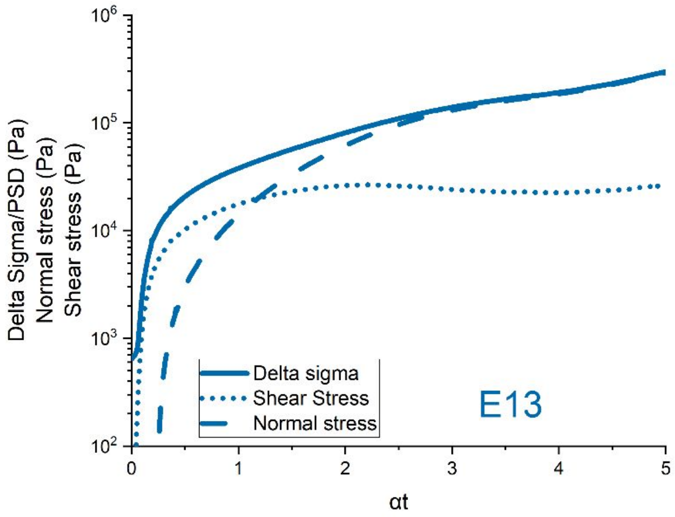

Figure S13: Polymer stress difference, normal stress, and shear stress for the sample E13.

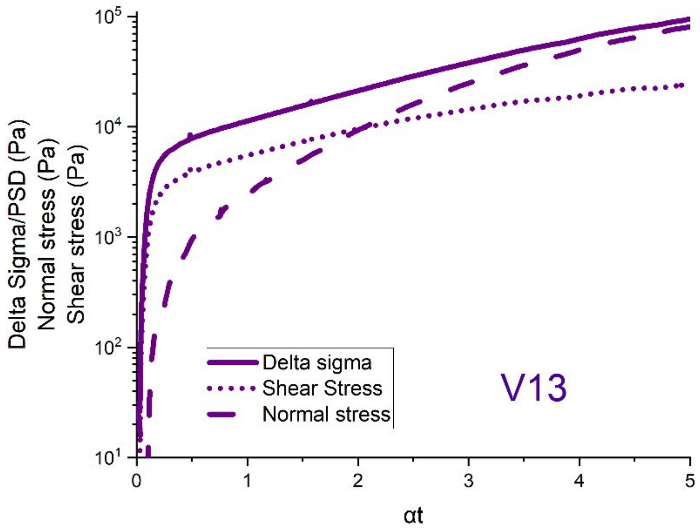

Figure S14: Polymer stress difference, normal stress, and shear stress for the sample V13.

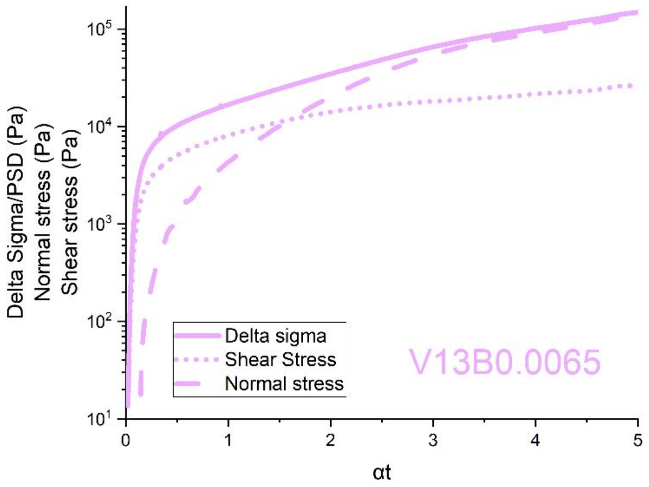

Figure S15: Polymer stress difference, normal stress, and shear stress for the sample V13B0.0065.
